# Assessing the Impact of Climate-Smart Agricultural Practices on Household Welfare and Poverty Among Smallholder Maize Farmers in KwaZulu-Natal Province, South Africa

**DOI:** 10.3390/foods15040694

**Published:** 2026-02-13

**Authors:** Minentle L. Mnukwa, Lelethu Mdoda, Yanga Nontu, Samuel S. Ntlanga, Phiwe Jiba, Lwando Mbambalala, Lungile S. Gidi, Mosima M. Mabitsela

**Affiliations:** 1 Discipline of Agricultural Economics, School of Agriculture and Science, University of KwaZulu-Natal, P/Bag X01, Scottsville, Pietermaritzburg 3209, South Africa; mdodal@ukzn.ac.za (L.M.);; 2 Department of Agriculture, University of Zululand, P/Bag X1001, KwaDlangezwa 3886, South Africa; nontuy@unizulu.ac.za (Y.N.);; 3 School of Interdisciplinary Research and Graduate Studies, College of Graduate Studies, University of South Africa (UNISA), Preller Street, Muckleneuk Ridge, Pretoria 0003, South Africa; 4 Department of Agricultural Economics and Animal Production, University of Limpopo, Private Bag X1106, Sovenga 0727, South Africa; lungile.gidi@ul.ac.za

**Keywords:** agricultural resilience, climate-smart agricultural practices, endogenous switching regression, food insecurity, household welfare, maize farmers, poverty, smallholders, South Africa

## Abstract

Climate-smart agricultural practices (CSAPs) are promoted as pathways for improving productivity and resilience among smallholder farmers; however, empirical evidence on their welfare effects remains limited in South Africa. This study examines the impact of CSAP adoption on household welfare among smallholder maize farmers in KwaZulu-Natal Province. A cross-sectional survey of 300 households was conducted using a multistage sampling approach. Welfare outcomes was measured using multidimensional indicators including the Household Dietary Diversity Score (HDDS), the Household Food Insecurity Access Scale (HFIAS), the Coping Strategy Index (CSI), and the Foster–Greer–Thorbecke (FGT) poverty index. An Endogenous Switching Regression (ESR) model was employed to correct for selection bias and to generate counterfactuals that estimate what adopters’ welfare would have been in the absence of CSAP uptake. Results show that access to extension, group membership, and training significantly increased the likelihood of CSAP adoption. ESR outcomes indicate that adopters had higher dietary diversity, lower food insecurity, and reduced reliance on severe coping strategies. Counterfactual analysis reveals that adopters would have experienced significantly poorer welfare outcomes had they not adopted CSAPs. The findings demonstrate that CSAP adoption yields measurable welfare benefits and improves household resilience. The study recommends targeted investments in extension support, farmer organizations, and institutional arrangements to accelerate the adoption of CSAP and enhance household welfare.

## 1. Introduction

Globally, agriculture remains one of the most climate-sensitive sectors, serving as the primary source of food, income, and livelihoods for billions of people [1]. According to [2], climate change risk to food, livelihoods, and income refers to the disruption and breakdown of agricultural and food systems (including crop, livestock, and fisheries production) as well as interruptions in food distribution networks. Global warming, recurrent droughts, floods, and increasing precipitation variability and extremes drive these challenges. Such impacts are particularly severe for populations already vulnerable due to low income, limited access to productive resources, social marginalization, or other structural inequalities that constrain their capacity to secure sustainable livelihoods [3]. However, climate change has increasingly undermined agricultural productivity, threatening global food systems and rural welfare [4]. Studies estimate that anthropogenic warming has reduced global agricultural total factor productivity by approximately 21% since 1961, with losses of 30–33% in warmer regions such as Africa and Latin America, even after accounting for partial offsetting benefits from CO_2_ fertilization effects on photosynthesis [5]. Climate change, coupled with rapid population growth, places the food and livelihood security of many people (particularly in developing countries) under increasing threat [6]. Key challenges affecting agricultural production include rising temperatures, declining and more erratic rainfall, and heightened incidences of pests and diseases.

Additionally, climate change is already compromising global water availability, contributing to more frequent and severe droughts and floods, which further exacerbate risks to food systems and livelihoods [7]. As the world faces a growing population, resource constraints, and environmental degradation, the interplay between climate change and agriculture poses serious challenges to sustainable development and food security [8]. Recent reports estimate that climate-related disasters caused global agricultural losses exceeding USD 3.8 trillion between 2008 and 2022, further demonstrating the scale of economic risk facing farming communities [9].

Across Africa, agriculture is the backbone of most economies, employing nearly 60% of the workforce and contributing significantly to national GDPs. In Africa, where livelihoods heavily depend on climate-sensitive natural resources, the changing climate poses serious threats to ecological systems, diminishing their productivity and resilience, and thereby constraining human development, livelihoods, and food availability [10,11]. Yet, the continent’s agricultural systems are predominantly rain-fed, making them highly vulnerable to erratic rainfall, droughts, heat stress, and other climatic shocks [12]. In Sub-Saharan Africa (SSA), yields of key staple crops, such as maize, sorghum, and millet, are projected to decline by 10–20% compared to what they would have been in the absence of climate change under moderate warming scenarios [5]. In SSA, it is projected that nearly 80% of the global population faces heightened risks of crop failure and famine due to the escalating impacts of climate change, which will reduce productivity [13]. Consequently, climate change not only threatens food production but also the livelihoods and welfare of smallholder farmers who depend on agriculture for survival. Persistent droughts, floods, and land degradation have already placed millions at risk of hunger and income insecurity [9]. Furthermore, approximately 282 million Africans experienced undernourishment (defined as prolonged inadequate food consumption to meet dietary energy requirements) in 2023, representing a 25 million increase compared to pre-pandemic levels [8]. This heightened vulnerability carries profound developmental implications for the continent, constraining economic growth, exacerbating poverty, and undermining the overall well-being of its people [10]. Given Africa’s rapid population growth, limited infrastructure, and constrained adaptive capacity, the urgency of climate-resilient agricultural transformation across the continent cannot be overstated [5].

In South Africa, and particularly in KwaZulu-Natal (KZN) Province, these challenges manifest with increasing severity. Smallholder farmers in the KZN Province play a vital role in food production and rural employment, but face mounting threats from climate-induced shocks [14,15]. The province has experienced intensified droughts, erratic rainfall, soil erosion, and temperature extremes, all of which negatively affect crop productivity and household welfare [16]. Over the past two decades, climate disruptions have become increasingly frequent, leading to crop failures, reduced yields, and heightened food insecurity in rural areas [17]. Moreover, climate risks like the recurring droughts, floods, and changes in water availability compromise the ability to irrigate crops and provide water for livestock [18]. Maize, being both a staple food and a key cash crop, stands at the centre of this challenge. It forms the backbone of household diets and rural economies, yet its production has been declining due to changing rainfall patterns, pest infestations, and declining soil fertility [19]. Historically, maize farming in the KZN Province has been a cornerstone of smallholder agriculture, particularly in regions such as uMkhanyakude, Zululand, and the Harry Gwala District, where it provides both subsistence and market-oriented benefits. However, declining productivity has weakened the role of maize as a reliable source of livelihood. Smallholders’ dependence on rain-fed production systems and limited access to agricultural inputs, technology, and climate information have deepened their vulnerability. Recent agricultural assessments indicate that more than 20% of households in KZN Province experience inadequate food access, and child malnutrition in rural districts remains above the national average [20]. Despite its agricultural potential, KZN Province remains one of the provinces with the highest levels of rural poverty and household food insecurity in South Africa, highlighting the urgent need for interventions that strengthen resilience and improve household welfare [20]. These structural challenges underscore the need for sustainable solutions that enhance resilience, productivity, and welfare outcomes.

In response, Climate-Smart Agriculture (CSA) has emerged as a transformative framework to address the twin challenges of productivity and resilience [21]. CSA seeks to (i) sustainably increase agricultural productivity and incomes, (ii) adapt and build resilience to climate change, and (iii) reduce or remove greenhouse gas emissions where possible [22]. Climate-Smart Agricultural Practices (CSAPs) (including conservation tillage, crop diversification, agroforestry, intercropping, organic manure application, and rainwater harvesting) offer viable pathways for achieving these goals. For this study, CSAPs are operationally defined as farming practices that simultaneously address productivity, adaptation, and mitigation objectives in line with the FAO’s climate-smart agriculture framework [22]. Specifically, this research examines four CSAPs widely promoted and accessible to smallholder farmers in KZN Province: (1) drought-tolerant maize varieties, improved hybrid or open-pollinated varieties with enhanced moisture stress tolerance and shorter growing periods suited to increasingly erratic rainfall patterns; (2) maize-legume intercropping, the simultaneous cultivation of maize with nitrogen-fixing legumes (cowpea, beans, or groundnuts) to improve soil fertility, diversify production, and enhance food security; (3) rainwater harvesting, in-field water conservation techniques including tied ridges, basins, and mulching practices that capture and retain soil moisture during dry spells; and (4) crop diversification, cultivation of three or more crop species beyond maize to spread climate risk and improve dietary diversity. According to literature and past studies, the selected four practices were based on their relevance to the study area’s semi-arid agro-ecological conditions, documented effectiveness in enhancing climate resilience [23,24], and accessibility to resource-constrained smallholder farmers. Farmers are classified as CSAP adopters if they implemented at least two of these practices during the 2022/2023 agricultural season, reflecting a meaningful level of climate-smart agriculture integration likely to generate measurable welfare impacts [25].

Empirical evidence across SSA shows that CSAPs can improve yields, enhance food security, and reduce poverty. However, their adoption and impacts are highly context-specific, shaped by socio-economic, institutional, and environmental factors [23,24]. A recent study further demonstrates that the adoption of multiple CSAPs leads to higher dietary diversity and greater income stability than adopting a single practice [25], suggesting that welfare benefits accumulate with increased adoption intensity.

In South Africa, although policies such as the National Climate Change Adaptation Strategy (NCCAS) and the Agricultural Green Economy Plan promote the integration of CSA into smallholder systems, empirical studies examining the welfare outcomes of CSAP adoption remain limited and fragmented. Most existing research has focused primarily on the determinants of adoption rather than its impacts on household welfare, food security, and poverty dynamics. This gap is particularly significant in KZN Province, where diverse agro-ecological zones, gendered farming systems, and dual land-tenure arrangements create unique contexts for adoption and impact assessment [26]. Furthermore, KZN Province continues to face persistent rural poverty and food insecurity despite its agricultural potential. Ref. [27] reports that a large proportion of rural households in the province lack access to productive assets, credit, irrigation infrastructure, and agricultural extension services. In response, several provincial initiatives, such as the Climate-Smart Agriculture Programme and the Farmer Support and Development Programme, have been introduced to strengthen input supply, farmer training, and technology dissemination. However, these interventions remain unevenly distributed across municipalities, and constraints related to extension capacity, market access, and institutional fragmentation continue to shape differential adoption outcomes [28,29]. Traditional coping strategies, such as asset liquidation and reliance on social grants, provide only short-term relief without addressing the underlying vulnerability to climate-induced shocks. Thus, improving access to CSAPs represents a strategic opportunity not only to build climate resilience but also to break persistent cycles of poverty and food insecurity in KZN Province [28]. What remains unclear, however, is whether the adoption of CSAPs truly improves household welfare, dietary diversity, and poverty outcomes, particularly when controlling for self-selection bias (i.e., that adopters are inherently different from non-adopters).

This study seeks to address the existing knowledge gap by rigorously assessing the impact of CSAPs on household welfare and vulnerability to poverty among smallholder maize farmers in KZN Province. In doing so, it moves beyond the conventional focus on adoption determinants to examine the broader welfare implications of climate-smart interventions within smallholder farming systems. Specifically, the study pursues three interrelated objectives: first, to explore the relationship between CSAP adoption and household food security, thereby evaluating how these practices contribute to improved dietary diversity, production stability, and resilience against climatic shocks; second, to analyze the role of CSAPs in reducing poverty and vulnerability, with particular emphasis on income enhancement, livelihood diversification, and asset accumulation; and third, to identify the socio-economic and institutional factors that shape the likelihood and intensity of CSAP adoption among smallholder maize farmers, including access to extension services, credit, markets, and information. Integrating counterfactual impact evaluation (ESR), multidimensional food security indicators (HFIAS, HDDS, CSI), and poverty indices (FGT), this study provides the causal empirical evidence on whether CSAPs improve the welfare of smallholder maize farmers in KZN Province. By integrating these analytical dimensions, the study aims to generate robust, evidence-based insights that inform policies and interventions promoting inclusive, climate-resilient, and welfare-enhancing agricultural systems. Ultimately, the findings are expected to support policymakers, development practitioners, and researchers in formulating strategies that strengthen adaptive capacity, foster sustainable livelihoods, and advance the broader agenda of food security and rural development in South Africa and across the African continent.

### Theoretical Framework of the Study

**Figure 1:**
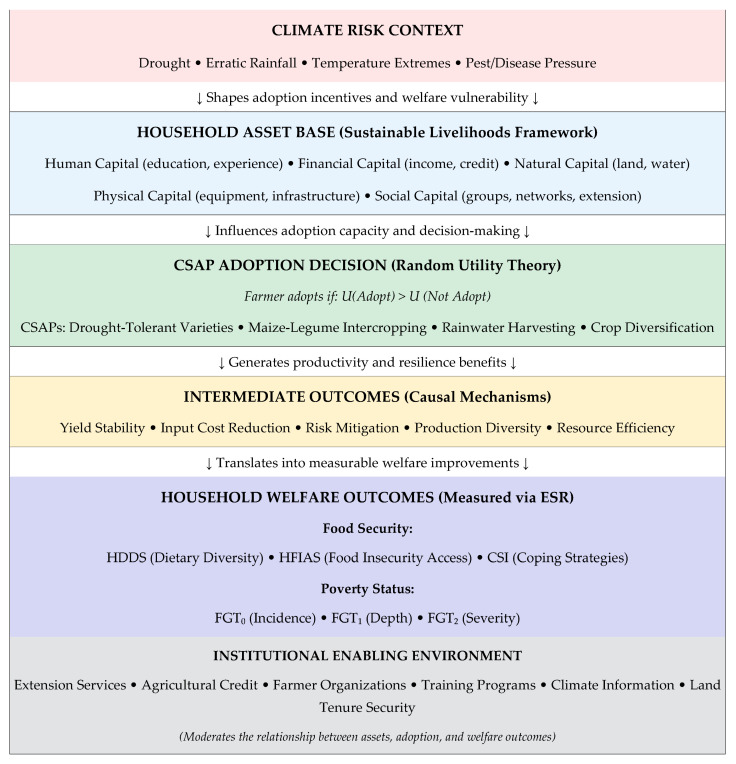
Integrated Theoretical Framework synthesizing Random Utility Theory (RUT) and the Sustainable Livelihoods Framework (SLF). The diagram synthesizes Random Utility Theory (RUT) and the Sustainable Livelihoods Framework (SLF) to illustrate the conceptual pathways from household assets and climate risk context through CSAP adoption decisions to welfare outcomes. Source: Authors’ conceptualization.

The adoption of CSAPs and their impact on household welfare can be best understood through the integration of theories (Figure 1) rooted in microeconomic decision-making and development economics. Smallholder maize farmers in rural South Africa operate in contexts characterized by resource constraints, imperfect information, and high exposure to climate-related shocks. Their adoption choices are not only shaped by expected benefits but also by perceived risks, transaction costs, and institutional factors [17]. Therefore, an appropriate theoretical framework should explain both the decision to adopt CSAPs and the subsequent impacts on welfare and poverty. The study adopted the Random Utility Theory (RUT) as its theoretical foundation.

The RUT provides a robust theoretical foundation for understanding maize farmers’ adoption of CSAPs in KZN Province. Rooted in microeconomic decision-making, RUT posits that individuals are rational agents who make choices among available alternatives to maximize utility [30]. However, because not all determinants of choice are observable, the utility derived from any option is modeled as a combination of a systematic (deterministic) component and a random (unobserved) component. This makes RUT particularly suitable for analyzing adoption decisions in uncertain environments such as smallholder farming systems in South Africa [29].

The Random Utility Model (RUM), originally formalized by [31], is a widely applied framework for analyzing individual choice behaviour, as it explicitly incorporates stochastic or latent factors that shape decision-making. One of its main strengths is its ability to capture unobservable components that traditional deterministic models often overlook [32]. Moreover, the RUM is firmly rooted in economic theory, thereby providing a robust analytical foundation for studying consumer preferences and decision processes [33]. In operational terms, the indirect utility within a typical RUM is specified as a linear function of the observable characteristics of the available alternatives, socio-demographic attributes of decision-makers, preference-related features, and a stochastic error term that accounts for unobserved heterogeneity.

In this study, smallholder maize farmers face a fundamental decision between adopting CSAPs or continuing with conventional farming methods. Each choice entails a combination of benefits, costs, and risks. While CSAP adoption has the potential to increase yields, stabilize production, and strengthen household food security, it may also introduce challenges such as higher labour demands, upfront investment costs, and uncertain market rewards. RUT provides a valuable foundation for analyzing this trade-off by assuming that farmers will adopt CSAPs only when the expected utility of adoption outweighs that of non-adoption, given their individual constraints and preferences. This framing captures the uncertainty and heterogeneity that characterize smallholder agricultural systems.

However, traditional RUT explanations are insufficient on their own when applied to smallholder farming contexts marked by climate variability and structural limitations. To address this, the study extends RUT by integrating three critical determinants (climate risks, institutional factors, and household resource endowments) that jointly influence the perceived utility and feasibility of adopting CSAPs. Climate risks such as drought, rainfall variability, and heat stress intensify production uncertainty, thereby increasing the expected utility of practices that promote resilience and reduce vulnerability. Institutional factors, including access to extension services, credit, farmer organizations, climate information, and training, help lower transaction costs and information asymmetries, thereby enhancing farmers’ capacity to adopt and maintain CSAPs. Similarly, household resource endowments (such as land size, labour availability, income, education, farming experience, and asset ownership) shape the farmer’s ability to invest in and sustain CSAP implementation. Together, these factors provide a comprehensive extension of RUT that more accurately reflects the complex decision-making processes of smallholder farmers under climate uncertainty.

The utility function in RUT is expressed as:

(1) $$\cup_{ij}=\;V_{ij}+\;\varepsilon_{ij}$$

where $\cup_{ij}$ represents the utility that maize farmer i derives from choosing alternative j (e.g., adopting or not adopting CSAPs), $V_{ij}$ is the observable (systematic) component influenced by measurable socio-economic, farmer characteristics and institutional variables, and $\varepsilon_{ij}$ is the unobservable (random) component [34]. A farmer will adopt CSAPs if:

(2) $$\cup_{i1}\;>\;\cup_{i0}=\;V_{i1}+\;\varepsilon_{i1}>V_{i0}+\;\varepsilon_{i0}$$

The probability of adoption can thus be represented as:

(3) $$P\;\left({Adopt}_{i}=1\right)=P\;\left(\cup_{i1}>\cup_{i0}\right)=P\;(V_{i1}-\;V_{i0}>\;\varepsilon_{i0}-\varepsilon_{i1})$$

This mathematical structure enables the study to model adoption decisions using empirical econometric techniques.

According to RUT, utility can be decomposed into two parts: a deterministic component influenced by observable household characteristics (e.g., income, farm size, education, access to extension services) and a stochastic component capturing unobserved factors such as risk attitudes, perceptions, and social influences. According to RUT, the systematic component of utility is often specified as a linear function of household characteristics:

(4) $$V_{ij}=\;X_{i}\beta_{j}$$

where $X_{i}$ is a vector of household and farm-level characteristics (such as land size, access to credit, extension services, and education), and $\beta_{j}$ is a vector of parameters to be estimated. The unobservable component $\varepsilon_{j}$ captures heterogeneity in preferences, risk attitudes, and other unmeasured influences. By decomposing utility in this way, RUT provides a framework that aligns seamlessly with econometric approaches such as the endogenous switching regression model used in this study, which corrects for selection bias and estimates counterfactual outcomes. This decomposition is essential because it allows the econometric analysis to account for both measurable socio-economic variables and unobservable heterogeneity that influence CSAP adoption [35]. The use of an endogenous switching regression model in this study is consistent with this framework, as it addresses the selection bias arising from unobserved characteristics influencing adoption. Within this RUT framework, the adoption decision triggers several mediating mechanisms (improved productivity, yield stability, income growth, asset accumulation, and enhanced food availability and dietary diversity) that collectively shape household welfare outcomes. These mediating channels explain how CSAP adoption translates into higher dietary diversity (HDDS), reduced food insecurity (lower HFIAS and CSI scores), and lower poverty levels. Thus, the framework provides a coherent causal pathway that links RUT-based adoption behaviour with welfare impacts under climate risk, offering a strong theoretical justification for the empirical estimation strategy applied in this study.

Beyond adoption, RUT also provides insights into welfare outcomes. The Random Utility Theory (RUT) forms the core of the study’s theoretical framework and is directly reflected in the structure of the conceptual diagram. RUT posits that smallholder maize farmers choose to adopt or not adopt CSAPs based on the option that offers the highest expected utility under conditions of climate uncertainty. As shown in the diagram, this decision is shaped by three major determinants: climate risks, institutional support, and household resource endowments. Heightened climate risks (such as droughts, rainfall variability, floods, and heat stress) increase production uncertainty and therefore raise the utility of adopting practices that stabilize yields and reduce vulnerability. At the same time, institutional factors such as extension services, credit access, farmer groups, and climate information, together with resource endowments like land, labour, income, assets, and education, influence a household’s ability to adopt CSAPs. These determinants jointly shape farmers’ perceived utility, explaining the directional arrows feeding into the RUT box in the figure and establishing the foundation for the adoption decision.

Once the RUT-based decision is made and CSAPs are adopted, the diagram illustrates how this triggers several mediating mechanisms, including productivity gains, yield stability, asset accumulation, improved food availability, and dietary diversification, through which utility gains are realized. Farmers adopt CSAPs not merely as technical adjustments but as strategies to maximize household wellbeing through consumption smoothing, reduced exposure to shocks, and enhanced resilience. RUT also supports the estimation of counterfactual scenarios by acknowledging that farmers are heterogeneous and make decisions under uncertainty, allowing the study to compare what adopters would experience without adoption and what non-adopters would experience with adoption. The bottom section of the diagram, household welfare outcomes (food security and poverty alleviation), captures the final impact of these mechanisms. Because food security and poverty indicators are direct reflections of household decision-making under risk, RUT provides a coherent explanation for why adoption leads to higher dietary diversity, lower food insecurity, reduced coping behaviours, and lower poverty levels. Grounding the study in RUT therefore creates a systematic and credible linkage between CSAP adoption, mediating pathways, and measurable welfare outcomes, strengthening both the empirical analysis and its policy relevance.

The RUT provides a strong theoretical lens for this research by explaining why and how smallholder maize farmers in KZN Province adopt climate-smart practices and how these choices influence household welfare and poverty vulnerability. Its focus on rational decision-making under uncertainty, incorporation of observable and unobservable factors, and compatibility with econometric modeling make it the most suitable theory for this study. By grounding the analysis in RUT, the research can systematically link CSAP adoption decisions to measurable welfare and poverty outcomes, thereby offering credible insights for both academic inquiry and policy formulation.

Sustainable Livelihoods Framework (SLF) with Climate-Smart Agricultural Practices (CSAPs) for smallholder maize farmers.

The Sustainable Livelihoods Framework (SLF) offers a comprehensive lens for understanding how smallholder farmers make decisions under conditions of risk and uncertainty, particularly in the context of climate-related risks. Developed by the UK’s Department for International Development (DFID), the framework emphasizes the interplay between household assets, vulnerability contexts, institutional structures, livelihood strategies, and outcomes [36,37,38]. The sustainable livelihood capital approach is a theoretical framework used in the field of sustainable development and poverty reduction [39]. Ref. [40] specified that many researchers within the development field have refined and expanded this approach through both research and practical application. It serves as a framework for examining the resources and assets available to individuals and communities, enabling them to enhance their living standards. Hence, in the context of smallholder maize farmers in KZN Province, SLF offers a holistic approach to analyze how households respond to climate variability, resource constraints, and institutional factors when deciding whether to adopt Climate-Smart Agricultural Practices (CSAPs). Unlike purely technical models, SLF captures the multidimensional nature of livelihoods, connecting environmental, social, economic, and institutional dimensions to farmer decision-making processes and outcomes.

Within SLF, the vulnerability context represents external shocks, trends, and seasonality that affect households’ livelihoods. For smallholder maize farmers, climate variability manifests as erratic rainfall, droughts, floods, and extreme weather events, directly impacting natural capital, crop yields, and food security. These climate risks create uncertainty in production outcomes, influencing farmers’ decisions regarding investment in or adoption of CSAPs, such as drought-tolerant maize varieties, conservation agriculture, or soil and water management practices. By framing climate hazards within the context of vulnerability, SLF enables researchers to assess how these risks interact with household resources and capacities to shape adaptive strategies, providing a structured approach to explore why some farmers adopt CSAPs while others remain constrained.

SLF emphasizes the role of five key livelihood assets (human, social, natural, physical, and financial capital) in shaping households’ capacity to pursue sustainable livelihood strategies. Examining how individuals and communities utilize, enhance, and create synergies among these capitals provides insight into the enabling conditions that support their adaptation to the specific environmental and social contexts they face [41]. Human capital, including education, knowledge of climate-smart practices, and farming experience, enhances adaptive decision-making. Social capital, such as networks, farm organizations or cooperatives, and community support, facilitates information sharing and collective action for CSA adoption. Natural capital, including land quality, available infrastructure, and access to water, determines the feasibility of implementing certain practices. In contrast, physical and financial capital influence the ability to invest in inputs, tools, or technologies. In the KZN Province, the differential availability and quality of these assets help explain the variation in adoption rates of CSAPs, with households possessing higher levels of capital being more likely to adopt strategies that improve resilience and productivity.

Transforming structures and processes (such as policies, institutions, market access, and extension services) mediate access to assets and shape the feasibility of livelihood strategies. Extension programs that provide training, information, and advisory support can increase knowledge and confidence in adopting CSAPs. At the same time, access to credit, subsidies, and input markets facilitates the implementation of resource-intensive practices. Within this framework, livelihood strategies encompass both conventional maize farming and adaptive responses, including diversification, crop-livestock integration, and CSA adoption. SLF enables the analysis of how these strategies are influenced not only by asset endowments and vulnerability contexts but also by the institutional environments that allow or constrain them, offering a nuanced understanding of decision-making pathways.

The ultimate focus of SLF is on livelihood outcomes (household welfare), which reflect the effectiveness of strategies in improving household welfare, reducing vulnerability, alleviating poverty, enhancing food security and income, and enhancing resilience. For smallholder maize farmers, successful adoption of CSAPs can lead to increased yields, greater food security, higher incomes, alleviate poverty, and reduce susceptibility to climate-induced shocks [42]. Conversely, households with limited assets or poor access to supportive institutions may experience lower adoption rates and remain vulnerable to poverty and climatic stress. Integrating SLF into the study of CSAPs provides a clear framework for identifying the factors that most influence adoption and outcomes, enabling policymakers and development practitioners to design interventions that strengthen assets, alleviate poverty, improve access to knowledge and credit, and build institutional support to promote sustainable livelihoods and climate resilience in KZN Province.

While RUT and SLF originate from different disciplinary traditions, microeconomics and development studies, respectively, their integration provides a more comprehensive analytical lens than either framework alone. RUT excels at explaining the decision-making process (why farmers choose to adopt CSAPs). At the same time, SLF provides the structural context (what resources, vulnerabilities, and institutional factors enable or constrain that decision). The complementarity between the two frameworks is evident at multiple levels. First, the five livelihood capitals in SLF (human, social, natural, physical, and financial) directly correspond to the observable characteristics (X_i_) in the RUT utility function. For instance, human capital shapes farmers’ ability to process information about CSAPs, financial capital determines their capacity to bear the costs of adoption, and social capital reduces transaction costs and information asymmetries. Second, the vulnerability context in SLF (climate shocks, market fluctuations, seasonal stress) aligns with RUT’s stochastic component (ε_i_ⱼ), capturing the uncertainty under which farmers make adoption decisions. Third, SLF’s transformation of structures and processes (extension services, credit institutions, and farmer organizations) corresponds to institutional factors that modify the deterministic utility component (V_i_ⱼ) and serve as exclusion restrictions in the ESR model. Finally, SLF’s livelihood outcomes map directly onto the welfare indicators measured in this study (HDDS, HFIAS, CSI, FGT indices). The integrated framework thus traces a complete causal chain: from livelihood assets and vulnerability context (SLF inputs), through utility-maximizing adoption decisions (RUT mechanism), to intermediate productivity effects, and ultimately to measurable welfare outcomes (SLF outputs). Figure 1 visually operationalizes this integration, depicting the interconnected pathways that link household assets, adoption decisions, and welfare outcomes under climate uncertainty.

## 2. Materials and Methods

### 2.1. Description of the Study Area

**Figure 2:**
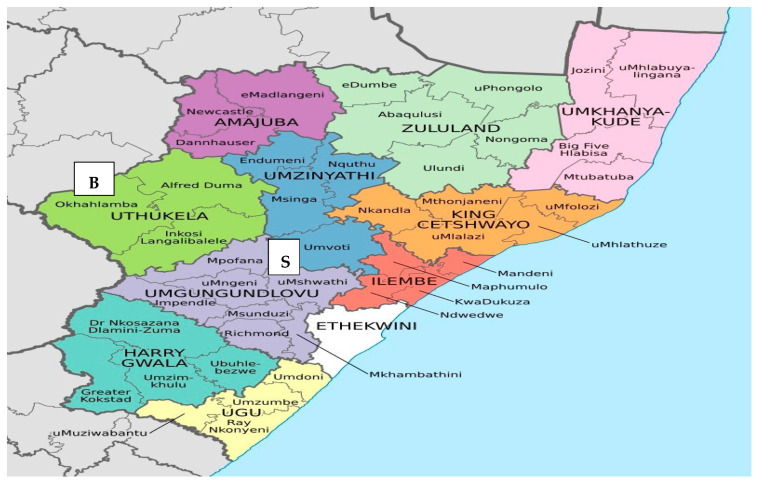
Map showing HGDM and UDM, in KZN Province of South Africa. All district municipalities labeled from “B” and “S” upwards in the figure are not study sites. Source: Municipal boundaries were obtained from the Municipal Demarcation Board of South Africa [47].

The study was conducted in KZN Province, South Africa, focusing on the Harry Gwala District Municipality (HGDM) and Ugu District Municipality (UDM) (Figure 2). The KZN Province was purposively selected due to its prominence in maize farming and its documented vulnerability to climate change, including floods and extreme heat, which affect smallholder agricultural productivity [27,43]. HGDM and UDM were selected as study sites due to their differing agro-ecological conditions and significance in smallholder maize production. HGDM, an inland district with a temperate climate and frequent frost, spans 10,386 km^2^ with 461,420 residents, while UDM lies along the eastern coastline, experiencing a subtropical environment, covering 5866 km^2^ with 722,484 residents [44]. Agriculture is the economic backbone in both districts, with maize serving as a key crop for food security and income [45]. Climate-related challenges, such as erratic rainfall and rising temperatures, have created an urgent need for climate-adaptive practices, making these areas ideal for assessing the adoption and impact of CSAPs [27,46].

### 2.2. Sampling Design

This study employed a cross-sectional quantitative research design to examine the relationship between CSAP adoption, household welfare, and vulnerability to poverty among smallholder maize farmers. In a cross-sectional design, data are collected from a population at a single point in time to analyze relationships between variables or differences across groups [48,49]. The design is both cost-effective and time-efficient, enabling the simultaneous examination of multiple socio-economic and institutional factors that influence adoption decisions. The quantitative approach enabled objective measurement and statistical testing of hypotheses, improving the generalizability and scientific rigor of the findings [50]. This design is widely applied in agricultural economics and rural development studies, aligning with recent South African empirical studies that examine CSAP adoption and household welfare outcomes [15,51].

### 2.3. Sampling Procedure

A multi-stage sampling approach was employed to ensure comprehensive coverage of smallholder maize farmers operating under heterogeneous agro-ecological and institutional conditions. This design is particularly suitable for dispersed rural farming populations, as it offers a structured method for reducing sampling error while maintaining statistical representativeness and practical feasibility in welfare impact studies [52]. In the first stage, HGDM and UDM were purposively selected based on explicit criteria encompassing climate gradients, soil heterogeneity, and market accessibility. HGDM represents temperate inland conditions with average annual rainfall of 700–900 mm, temperature extremes ranging from −5 °C to 35 °C, and predominantly shallow acidic soils (Mispah and Glenrosa forms), while UDM features subtropical coastal conditions with 900–1100 mm rainfall, moderate temperatures (10 °C to 32 °C), and deeper alluvial soils (Inanda and Shortlands forms) [44]. Market accessibility also differs substantially: HGDM comprises remote mountainous areas with average distances of 45–60 km to urban markets, whereas UDM benefits from proximity to the Durban-Pietermaritzburg economic corridor with average market distances of 25–40 km [45]. Both districts exhibit high vulnerability to climate-induced shocks, including droughts, floods, and hail events, making them strategically important for evaluating CSAPs [27,46]. From each district, two local municipalities were purposively selected to represent intra-district variation: Greater Kokstad and Ubuhlebezwe from HGDM, and Umdoni and uMzumbe from UDM. The sampling frame was constructed using official farmer registers maintained by the KwaZulu-Natal Department of Agriculture and Rural Development (DARD), integrating extension officer registers updated as of January 2023, provincial smallholder databases from the 2020–2022 agricultural census, and membership rosters from registered farmer cooperatives [45]. Farmers were included if they cultivated maize as their primary activity (≥50% of land), farmed ≤5 hectares, resided in the study area for at least three consecutive seasons, and were actively engaged in farming. The combined sampling frame comprised 5068 eligible smallholder maize farmers (HGDM: 2734; UDM: 2334). In the subsequent stage, the sampling frame was stratified by CSAP adoption status to ensure adequate representation of both adopters and non-adopters for comparative analysis. Farmers were classified as adopters if they had implemented at least two of the following CSAPs during the 2022/2023 season: drought-tolerant maize varieties, maize-legume intercropping, rainwater harvesting, or crop diversification. Following sample size determination (see Section 2.4), the calculated sample of 300 households was proportionately allocated across districts and adoption status, yielding 129 CSAP adopters and 171 non-adopters.

In the final stage, systematic random sampling was applied within each stratum. Farmers were arranged alphabetically, a sampling interval (k) was calculated as the ratio of the stratum size to the desired sample size, a random starting point between 1 and k was selected, and every kth farmer was selected [52]. To address non-response bias, up to three contact attempts were made for each selected household at different times and days. Of 300 farmers initially selected, 15 could not be reached or declined participation (95% response rate), and replacements were randomly chosen from the same strata. Statistical tests comparing initial respondents and replacements revealed no significant differences in age, gender, farm size, or municipality (*p* > 0.10 for all comparisons). Sensitivity analysis comparing early respondents (those contacted first) with late respondents (those contacted second or third) showed no significant differences in CSAP adoption rates (*p* = 0.42) or welfare indicators (*p* > 0.15), confirming minimal non-response bias. Sample representativeness was validated against provincial agricultural statistics: sample mean farm size of 1.7 ha matched the population mean of 1.8 ha (*p* = 0.31), female-headed households comprised 44% versus 42% in the population (*p* = 0.58), and the sample adoption rate closely matched population estimates (*p* = 0.96), demonstrating that the sampling procedure successfully generated a representative sample supporting the external validity of the findings

### 2.4. Sample Size

To determine the appropriate sample size, the formula proposed by Yamane [53] was applied, which is widely used in studies with small finite populations to minimize sampling error. The formula is expressed as shown in Equation (1).

(5) $$n=\frac{N}{1+Ne^{2}}$$

where *n* is the sample size, *N* is the total population, and *e* is the desired level of precision. Based on a population of 5068 smallholder farmers obtained from provincial agricultural databases and a 5% margin of error, the estimated sample size was 300 households. This sample size was considered adequate to achieve statistical reliability and ensure representativeness of the population, consistent with guidelines by [54].

### 2.5. Data Collection

Primary data were collected using a structured questionnaire administered through face-to-face interviews with 300 smallholder maize farmers across HGDM and UDM. The instrument was designed to obtain information on four core domains: (i) demographic and socio-economic characteristics, (ii) farm production and resource endowment, (iii) access to institutional and support services (extension, credit, training), and (iv) adoption of CSAPs and household welfare indicators (HFIAS, HDDS, CSI). The questionnaire consisted mainly of closed-ended questions to ensure quantitative comparability, with a few open-ended items allowing respondents to elaborate on context-specific experiences and constraints. Pre-testing was conducted with 30 respondents (10% of the sample size) one month before actual data collection in Msunduzi Local Municipality, supervised by research supervisor Dr. L. Mdoda, who reviewed the results for validity, reliability, question clarity, and survey duration optimization, consistent with the recommendation that pre-testing enhances internal validity in survey-based studies [55]. The pre-testing results were excluded from the final analysis to maintain research integrity [56]. Construct reliability was further assessed using Cronbach’s alpha, where coefficients of 0.70 or higher were deemed acceptable [55]. To accommodate linguistic diversity, the questionnaire was translated into isiZulu and back-translated to ensure conceptual equivalence. Data were collected over 15 days by trained enumerators familiar with agricultural survey protocols. Enumerators underwent detailed training on research ethics, interviewing techniques, questionnaire administration, and data quality assurance procedures. All interviews were conducted in respondents’ homesteads and lasted an average of 45–60 min, allowing clarifications and minimizing misinterpretation among farmers with varying literacy levels.

Field challenges included difficulty locating households, weather disruptions, and respondent fatigue. These issues were mitigated through planning with local extension officers, flexible scheduling, and clear communication of research objectives, which enhanced trust and participation among stakeholders. Ethical clearance for this study was obtained from the University of KwaZulu-Natal Humanities and Social Sciences Research Ethics Committee (protocol number: HSSREC/00007887/2024). Participation was voluntary, and informed consent (English or isiZulu) was obtained from all respondents before data collection. The questionnaire explicitly captured adoption of four CSAPs relevant to smallholder maize systems in KZN Province: drought-tolerant maize varieties, maize–legume intercropping, rainwater harvesting, and crop diversification, enabling the subsequent ESR analysis of welfare outcomes between adopters and non-adopters.

### 2.6. Data Analysis

The data analysis employed a combination of descriptive and econometric techniques to assess the impact of CSAP adoption on household welfare and poverty vulnerability. Descriptive statistics, including means, standard deviations, percentages, *t*-tests, and chi-square tests, were used to summarize and compare the demographic and socio-economic characteristics of adopters and non-adopters. For impact evaluation, an Endogenous Switching Regression (ESR) model was employed to address selection bias arising from the non-random adoption of CSAPs [57]. The first stage estimated the probability of adoption using a probit model, while the second stage estimated welfare outcomes for adopters and non-adopters. The ESR model’s advantage lies in its ability to generate counterfactual outcomes, allowing comparison of what adopters’ welfare would have been without CSAP adoption, and vice versa [58]. Welfare indicators included the Household Food Insecurity Access Scale (HFIAS), Household Dietary Diversity Score (HDDS), and Coping Strategy Index (CSI). At the same time, poverty was measured using the Foster-Greer-Thorbecke (FGT) indices. Model diagnostics such as the Wald chi-square and lambda coefficients were used to test for model fitness and the presence of selection bias. All analyses were performed using Stata (Version 18, StataCorp LLC, College Station, TX, USA), ensuring robustness and reproducibility of the results.

#### 2.6.1. Household Dietary Diversity Score (HDDS)

Table 1 presents the twelve food groups used to compute the HDDS, where households scored one point for each food group consumed within the previous 24 h. The HDDS was used to measure diet quality as a proxy for the utilization dimension of household food security. It reflects the number of food groups consumed by the household within the previous 24 h, based on the assumption that greater dietary diversity is associated with better nutrient adequacy and improved welfare outcomes [59]. A 24 h recall period was used because it minimizes recall bias and reduces respondent burden. Each food group consumed scored 1 point, giving a possible HDDS range of 0–12, where higher scores indicate better dietary diversity. Following [60], HDDS scores were categorized into three levels: Low (1–4), Medium (5–8), and High (9–12).

#### 2.6.2. Household Food Insecurity Access Scale (HFIAS)

Table 2 lists the nine standardized HFIAS occurrence questions used to capture households’ behavioural and experiential indicators of food insecurity over the previous 30 days. The HFIAS was used to measure the access dimension of food security by capturing behavioural and experiential indicators of food insecurity within the past 30 days [59]. The scale contains nine occurrence questions and nine follow-up frequency questions. Responses are coded as: 0 = Never, 1 = Rarely (1–2 times), 2 = Sometimes (3–10 times), 3 = Often (>10 times). The total HFIAS score ranges from 0 to 27 with higher scores indicating greater food insecurity. Households are then categorized into four groups: food-secure, mildly food-insecure, moderately food-insecure, or severely food-insecure.

#### 2.6.3. Coping Strategy Index (CSI)

Table 3 summarizes the coping behaviours and their assigned severity weights used to calculate the CSI, where higher scores indicate greater food insecurity. The CSI was applied to assess household vulnerability by quantifying how often and how severely households resort to coping strategies when food is limited. The index incorporates both the frequency and severity weight of coping behaviours [61,62].

The CSI is calculated as:

$$CSIi=\sum ({frequency}_{is}\times {severity\;weights}_{s})$$

where higher CSI values reflect greater food insecurity and heavier reliance on negative coping mechanisms (e.g., skipping meals, reducing meal sizes, reducing adult consumption for children).

### 2.7. Econometric and Analytical Model Equations

This section presents the key analytical and econometric equations applied in the study to assess the impact of CSAPs on household welfare and vulnerability to poverty among smallholder farmers in KZN Province, South Africa.

#### 2.7.1. Foster-Greer-Thorbecke Poverty Indices

The Foster-Greer-Thorbecke index measures the incidence, depth, and severity of poverty. The general formula is expressed as:

$$FGT_{a}=\;(1/N)\;\Sigma \;[(z\;-\;y_{i})/z{]}^{a}\;I\;(y_{i}<z)$$

where

- z is the poverty line
- $y_{i}$ is the income of household i
- N is the sample size
- I ${(y}_{i}$ < z) = 1 if household is poor, 0 otherwise

When α = 0, it measures the headcount ratio (poverty incidence);

α = 1 measures the poverty gap (depth);

α = 2 measures poverty severity.

#### 2.7.2. Food Security Indices

(a) Household Dietary Diversity Score (HDDS):

$${\mathrm{HDDS}}_{i}=\Sigma \;F_{i}g$$

where $F_{i}$g = 1 if household i consumed food group g, 0 otherwise.

The HDDS is computed as the sum of food groups consumed by a household over a reference period. Higher HDDS values indicate greater dietary diversity.

(b) Household Food Insecurity Access Scale (HFIAS):

$${\mathrm{HFIAS}}_{i}=\Sigma \;q_{i}k$$

where $q_{i}$k is the response code for question k (0 = never, 1 = rarely, 2 = sometimes, 3 = often).

The HFIAS is the total score from frequency-based food access questions. Lower scores indicate higher food security.

(c) Coping Strategy Index (CSI):

$${\mathrm{CSI}}_{i}=\Sigma \;w_{s}\;f_{is}$$

where w_s_ is the severity weight for strategy s and f_is_ is the frequency of use. The CSI combines both the frequency and severity of coping strategies used by households during food shortages.

#### 2.7.3. Endogenous Switching Regression (ESR) Model

According to [28] the ESR model estimates the welfare outcomes of adopters and non-adopters of CSAPs while correcting for selection bias due to non-random adoption decisions.

(a) Probit Model:

$$D_{1}^{*}=X_{iY}+u_{i}$$

$$D=1\;\mathrm{if}\;D_{1}^{*}>0\text{;}\;\mathrm{otherwise}\text{,}\;D=0$$

where D represents the binary adoption decision (1 = adopter, 0 = non-adopter), X_i_ is a vector of explanatory variables, and u_i_ is the error term.

Inverse Mills Ratios (IMR):

$$\lambda_{1i}=\;\varphi (X_{iY})/\phi (X_{iY})\;\mathrm{for}\;\mathrm{adopters}$$

$$\lambda_{0i}=\varphi (X_{iY})/[1-\phi (X_{iY})]\;\mathrm{for}\;\mathrm{non}\text{-}\mathrm{adopters}$$

(b) Outcome Equations:

$$\mathrm{For}\;\mathrm{adopters}\;(D=1):\;y_{1i}=X_{i}\beta_{1}+\sigma_{1}\lambda_{1i}+\varepsilon_{1i}$$

$$\mathrm{For}\;\mathrm{non}\text{-}\mathrm{adopters}\;(D=0):\;y_{0i}=X_{i}\beta_{0}+\sigma_{0}\lambda_{0i}+\varepsilon_{0i}$$

where y_i_ represents the welfare outcome (e.g., HFIAS, HDDS), β_1_ and β_0_ are parameter vectors, σ_1_ and σ_0_ capture the covariance between the selection and outcome equations, and λ_1__i_ and λ_0__i_ are the Inverse Mills Ratios that correct for selection bias.

The ESR model eliminates sample selection bias by incorporating Inverse Mills Ratios (λ_1__i_ and λ_0__i_) into the outcome equations, which serve as selection correction terms. These ratios, computed from the first-stage probit model, capture the correlation between unobserved factors influencing adoption decisions and unobserved factors affecting welfare outcomes [56]. The covariance parameters σ_1_ and σ_0_ measure the degree of selection bias: if significantly different from zero, they indicate that ordinary regression methods would yield biased treatment effect estimates. By explicitly modeling this correlation, the ESR framework provides unbiased estimates of CSAP impacts on household welfare, even when adoption is endogenous and farmers self-select based on characteristics that also independently influence welfare outcomes [55]. The model’s validity is assessed through diagnostic tests, including the Wald test for joint independence of the selection and outcome equations, where rejection of the null hypothesis (ρ = 0) confirms the presence of selection bias and justifies the ESR approach over conventional methods [28,56]. Furthermore, the ESR framework generates counterfactual outcomes that estimate what adopters would have experienced had they not adopted and what non-adopters would have experienced had they adopted, enabling rigorous causal inference in non-experimental settings [57].

#### 2.7.4. Treatment Effects and Counterfactuals

The ESR model generates counterfactual outcomes to estimate how adopters would perform if they had not adopted and vice versa. The expected values are computed as follows:

- E [$Y_{1i}$ I D = 1] = $X_{i}\beta_{1}$+ $\sigma_{1}\lambda_{1i}$ (Actual outcome for adopters)
- E [$Y_{0i}$ I D = 1] = $X_{i}\beta_{0}$+ $\sigma_{1}\lambda_{1}$cf (Counterfactual outcome for adopters)
- E [$Y_{0i}$ I D = 0] = $X_{i}\beta_{0}$+ $\sigma_{1}\lambda_{0i}$ (Actual outcome for non-adopters)
- E [$Y_{1i}$ I D = 0] = $X_{i}\beta_{1}$+ $\sigma_{1}\lambda_{0}$cf (Counterfactual outcome for non-adopters)

From these, the treatment effects are derived as:

- ATT E [$Y_{1i}$ I D = 1] − E [$Y_{0i}$ I D = 1]
- ATU E [$Y_{1i}$ I D = 0] − E [$Y_{0i}$ I D = 0]
- ATE E [$Y_{1i}$] − E [$Y_{0i}$]

## 3. Results and Discussion

### 3.1. Description of the Explanatory Variables Used in the Endogenous Switching Regression Model and Their Expected Outcomes

Table 4 presents the explanatory variables included in the analytical models to assess the impact of CSAPs on household welfare and vulnerability to poverty among smallholder maize farmers in KZN Province. The variables are categorized into demographic characteristics, socio-economic factors, farm characteristics, and institutional factors. These variables are hypothesized to influence both the adoption of CSAPs and the resulting welfare outcomes, including food security (measured by HFIAS, HDDS, and CSI) and poverty vulnerability (measured by Foster, Greer, and Thorbecke indices).

### 3.2. Demographic Characteristics of the Sampled Smallholder Maize Farmers in KwaZulu-Natal Province

Table 5 presents the demographic and socio-economic characteristics of adopters and non-adopters. The demographic and socio-economic characteristics of the 300 smallholder maize farmers sampled in KZN Province reveal significant differences between CSAP adopters (43%, *n* = 129) and non-adopters (57%, *n* = 171). The sample demonstrates a female-dominated farming population with 65% female participation. This pattern is consistent across both groups. These results align with findings by [27], who reported that women constitute the majority of smallholder farmers in the KZN Province. Key demographic disparities emerge between adopters and non-adopters. Adopters are substantially younger (mean 46 years vs. 58 years) and more educated (mean 10 vs. 8 years of schooling). These findings indicate that age and education play crucial roles in CSAP adoption decisions. Ref. [63] emphasized that younger, more educated farmers demonstrate greater receptivity to CSAPs. They have enhanced risk tolerance and better information processing capabilities. The overall age profile indicates a mature farming population, with an average age of 53 years (SD = 16). This suggests extensive agricultural experience but potential resistance to adopting new practices.

Economic characteristics reveal substantial disparities between the two groups. Adopters demonstrate significantly higher household incomes (ZAR 13,000 vs. ZAR 8000 per month). They also have higher employment rates (65% vs. 51% formal employment). This confirms that financial capacity remains a critical determinant of CSAP adoption. This income differential aligns with findings by [64], who identified financial constraints as the primary barrier to CSAPs among South African smallholder farmers. Household composition reveals that adopters tend to maintain larger households (7 members vs. 6). This potentially provides enhanced labour capacity for CSAP implementation. However, both groups face constraints due to limited landholdings, averaging 2 hectares. Farming experience averages 20 years across the sample. Non-adopters demonstrate slightly more experience (21 years) compared to adopters (19 years). These results suggest that extensive farming experience alone does not necessarily translate into the adoption of CSAP. It may sometimes represent resistance to changing established practices.

The most striking differences emerge in patterns of institutional access. Adopters consistently demonstrate superior access across all support systems. Agricultural group membership shows the most substantial disparity. About 91% of adopters belong to farmer organizations compared to just 34% of non-adopters. These results highlight the crucial role of social capital and collective learning mechanisms in facilitating the adoption of CSAP. Adopters also exhibit substantially better access to agricultural credit (72% vs. 41%). They have more training opportunities (51% vs. 26%) and better access to extension services (78% vs. 55%). Climate information access remains relatively high for both groups (84% vs. 63%). Land ownership patterns reveal that 81% of adopters own their land compared to 64% of non-adopters. These results indicate that tenure security encourages long-term investments in sustainable practices. Ref. [65] documented weak extension services and limited credit access as significant constraints to agricultural innovation in South Africa. These institutional gaps, combined with demographic and economic disparities, explain why adoption rates remain moderate despite high awareness levels. These results support the theoretical framework by [66], who argued that successful CSAP adoption requires convergence of awareness, resources, and institutional support.

### 3.3. Climate-Smart Agriculture Practices Adoption Patterns in KwaZulu-Natal Province

The analysis of CSAP adoption patterns among smallholder maize farmers in KZN Province reveals diverse uptake rates across the four main CSAPs examined in this study. The overall adoption pattern shows that 129 farmers (43%) have adopted at least one CSAP, while 171 farmers (57%) remain non-adopters. Among the specific practices, drought-tolerant maize varieties demonstrate the highest adoption rate at 42%, followed by maize-legume intercropping at 35%, rainwater harvesting at 28%, and crop diversification at 24%. This adoption hierarchy reflects farmers’ prioritization of practices that offer immediate productivity benefits and require relatively lower initial investments. The higher adoption of drought-tolerant varieties aligns with the increasing frequency of drought episodes in KZN Province and farmers’ recognition of the direct yield protection benefits these varieties offer. These results are consistent with [27], who noted that drought-tolerant varieties are often the first CSAP adopted by smallholder farmers due to their compatibility with existing production systems and clear yield benefits during stress conditions.

**Figure 3:**
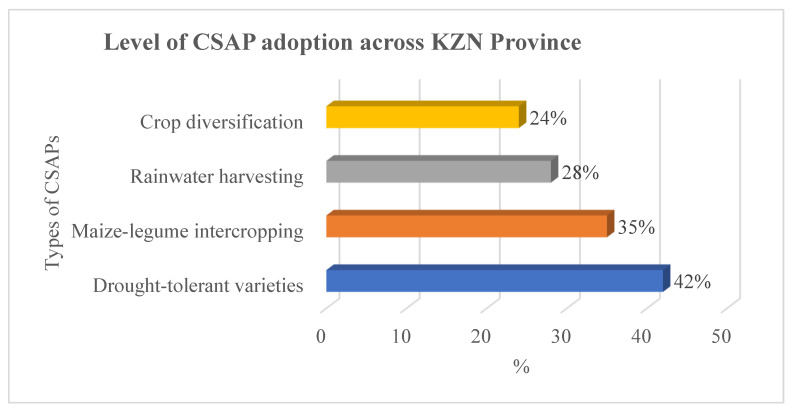
Types of climate-smart agriculture practices adopted and their distribution (%) among smallholder maize farmers (N = 300). Adoption rates: Drought-tolerant varieties 42%, Maize-legume intercropping 35%, Rainwater harvesting 28%, Crop diversification 24%. Chi-square test for distribution differences: χ^2^ = 23.47, df = 3. Source: Survey data generated through Stata version 18 (2025).

The adoption patterns (Figure 3) reveal essential insights about farmer decision-making regarding CSAPs in KZN Province. The preference for drought-tolerant varieties and intercropping suggests that farmers prioritize practices offering immediate, tangible benefits with relatively low implementation barriers. Ref. [67] noted that these practices require minimal changes to existing farming systems while providing clear advantages in terms of yield stability and input cost reduction. In contrast, the lower adoption rates for rainwater harvesting and crop diversification indicate that practices requiring significant system changes, higher investments, or specialized knowledge face greater adoption challenges despite their potential long-term benefits. Multiple CSAP adoption analysis reveals that 48% of adopters practice only one CSAP, 31% combine two practices, 16% use three practices, and only 5% implement all four CSAPs examined. This step-by-step adoption pattern suggests that farmers begin with simpler practices before progressing to more comprehensive climate-smart systems. Recent systematic reviews confirm this approach, showing that extension programs targeting integrated CSAP packages can maximize synergistic benefits while addressing farmers’ capacity constraints [68]. The geographical variations within KZN Province reflect farmers’ rational responses to location-specific climate risks, supporting the utility-maximizing framework. These findings align with recent evidence demonstrating that intercropping systems enhance food security and reduce input costs through biological nitrogen fixation, making them particularly attractive to smallholder farmers [69].

### 3.4. Food Security Analysis Results

#### 3.4.1. Household Food Insecurity Access Scale (HFIAS) Analysis

The following descriptive analyses (Section 3.4 and Section 3.5) present comparisons of food security and poverty indicators between CSAP adopters and non-adopters. However, as demonstrated in Table 5, these groups differ significantly in baseline characteristics; adopters are younger, more educated, have higher incomes, and better institutional access. Therefore, observed differences in welfare outcomes cannot be attributed solely to CSAP adoption, as they may reflect pre-existing disparities. These descriptive results provide contextual information but should be interpreted with caution. The causal impact of CSAP adoption on welfare outcomes is rigorously assessed in Section 3.6 through Endogenous Switching Regression, which controls for selection bias and generates counterfactual estimates that isolate the actual treatment effects.

Table 6 presents the comparative HFIAS outcomes by adoption status. The HFIAS analysis reveals substantial differences in food security status between CSAP adopters and non-adopters among smallholder maize farmers in KZN Province. The chi-square test (χ^2^ = 47.82, *p* < 0.001) indicates that the distribution of smallholder farmers across the four HFIAS categories differs significantly between adopters and non-adopters, confirming that adoption status and food security categories are statistically dependent rather than randomly distributed. This highly significant result validates that the observed differences in food security levels between the two groups are genuine and not due to chance.

The HFIAS results show that CSAP adopters have lower food insecurity scores compared to non-adopters, with adopters recording a mean HFIAS score of 7.8 versus 11.4 for non-adopters (*p* < 0.001). However, this 3.6-point difference cannot be directly attributed to CSAP adoption, given the significant baseline differences between groups documented in Table 5. ESR analysis was used to estimate the causal effect of CSAP adoption on food insecurity in Section 3.6, which controls for selection bias and provides unbiased treatment effect estimates. These findings align with evidence from Ethiopia, showing that CSA adopter households demonstrated significantly better food consumption scores, dietary diversity scores, and lower Food Insecurity Experience Scale scores compared to non-adopters [70]. The categorical analysis reveals striking differences between the two groups. Notably, 5% of adopters achieved complete food security (HFIAS = 0) compared to zero non-adopters, while 66% of adopters experienced only mild food insecurity, versus 33% of non-adopters. More importantly, the proportion experiencing severe food insecurity drops dramatically from 10% among non-adopters to only 2% among adopters. The moderate food insecurity category shows the most pronounced shift, with 57% of non-adopters falling into this category compared to just 27% of adopters. Ref. [71] demonstrated that this is consistent with recent research showing that CSAPs directly enhance agricultural productivity and food security, with food security being identified as the most crucial benefit driving farmers’ adoption decisions. These results provide strong evidence that CSAPs significantly enhance household food access and reduce the vulnerability to food insecurity among smallholder farmers, with the substantial reduction in severe food insecurity cases demonstrating the protective capacity of CSAPs against food system shocks. These findings align with recent evidence from similar contexts in SSA, where CSAPs have been shown to substantially improve food access outcomes through enhanced productivity and resilience mechanisms [15,70].

#### 3.4.2. Household Dietary Diversity Score (HDDS) Analysis

Table 7 presents the HDDS results by adoption status. The dietary diversity analysis reveals pronounced differences between CSAP adopters and non-adopters, indicating that CSAPs have a significant impact on diet quality among smallholder farming households. The chi-square test (χ^2^ = 34.67, *p* < 0.001) reveals that the distribution of households across the three HDDS categories differs significantly between adopters and non-adopters, indicating a strong association between adoption status and dietary diversity levels. This statistically significant result confirms that the observed differences in dietary diversity patterns between the two groups are not due to random variation but reflect genuine associations between CSAP adoption and improved diet quality.

The HDDS analysis reveals that CSAP adopters achieve markedly higher dietary diversity scores (7.2) compared to non-adopters (5.1), representing a substantial 41% improvement in dietary quality. This 2.1-point difference is both statistically highly significant (*p* < 0.001) and practically meaningful, representing an increase in consumption from approximately two additional food groups among adopting households. The categorical distribution demonstrates pronounced improvements across all dietary diversity levels. The proportion of households with low nutritional diversity decreases significantly, from 48.0% among non-adopters to 17.8% among adopters. Conversely, 23.3% of adopters achieve high dietary diversity, compared to just 7.6% of non-adopters, representing a more than threefold increase. The majority of adopters (58.9%) fall within the medium dietary diversity category, indicating that most adopting households achieve acceptable levels of dietary quality.

These findings align with recent empirical studies demonstrating similar positive impacts of CSAPs on dietary diversity. A study by [63] confirmed that adopters of conservation agriculture combined with crop diversification and small-scale irrigation showed statistically significant improvements in dietary diversity scores compared to non-adopters. A comprehensive study across SSA demonstrated that everyday climate adaptation practices, including tree management and home gardening, consistently enhanced Household Dietary Diversity Scores [72].

#### 3.4.3. Coping Strategy Index (CSI) Analysis

Table 8 presents the Coping Strategy Index (CSI) results by CSAP adoption status. The CSI measures household food insecurity by examining the frequency and severity of strategies households use when facing food shortages. Higher CSI scores indicate greater food insecurity as smallholder farmers rely more on harmful coping strategies. The analysis uses an independent samples *t*-test to compare mean CSI scores between adopters and non-adopters, testing whether the difference is statistically significant.

The CSI results reveal that CSAP adopters rely substantially less on negative coping strategies compared to non-adopters, with mean CSI scores of 14.7 versus 22.3, respectively (*p* < 0.001). This 7.6-point difference represents a 34% reduction in reliance on coping strategies, indicating that CSAPs provide a significant buffer against food system shocks and reduce the need for households to employ potentially harmful coping mechanisms.

The frequency analysis reveals significant reductions in the use of severe coping strategies among adopters. Most notably, 74.3% of non-adopters report “often” reducing meal portions compared to only 38.0% of adopters—representing a 49% reduction. Similarly, the practice of skipping entire meals drops from 45.0% among non-adopters to 18.6% among adopters. The most concerning coping strategy, reducing adult food intake to prioritize children, is employed by 50.9% of non-adopters, compared to 22.5% of adopters, representing a more than 55% reduction. Similarly, a study by [66] found that CSAPs significantly reduced household vulnerability and improved food security outcomes during climate shocks.

However, the relationship between CSAPs and coping strategies is not uniformly positive across all contexts. Research from India during the COVID-19 pandemic revealed that while crop diversity provided some protection against dietary diversity decline, the benefits were context-dependent and diminished when market access was severely restricted [67]. Additionally, a study examining rainwater harvesting systems in semi-arid regions found that the majority of farmers reported limited direct benefits from such interventions, with effectiveness declining significantly with distance from infrastructure [68]. It is worth noting that rainwater harvesting in smallholder contexts typically serves domestic water needs rather than irrigation; its benefits for food security are often indirect, operating through reduced time spent fetching water, which frees household labour for agricultural activities and other productive tasks. Nevertheless, these findings suggest that CSAP adoption alone may not guarantee a reduction in food insecurity for all households. Complementary investments in infrastructure and institutional support remain essential. These contradictory findings underscore the importance of complementary factors, such as market access, proximity to infrastructure, and broader support systems, in determining the effectiveness of CSAPs in reducing household vulnerability to food shocks.

### 3.5. Poverty Analysis Using Foster, Greer, and Thorbecke (FGT) Indices

The poverty analysis employed the Foster, Greer, and Thorbecke (FGT) indices to provide a comprehensive assessment of poverty dimensions among smallholder maize farmers based on their CSAP adoption status. The FGT indices capture three critical aspects of poverty: incidence (FGT_0_), depth (FGT_1_), and severity (FGT_2_), using the national poverty line of ZAR 760 per capita per month [69]. Table 9 presents the FGT poverty measures by adoption status.

#### 3.5.1. Poverty Incidence Analysis (FGT_0_)

The poverty headcount analysis reveals substantial differences in poverty incidence between the two groups. Among non-adopters, 68.2% of smallholder farmers (117 out of 171) fall below the national poverty line compared to 42.7% of CSAP adopters (55 out of 129), representing a 25.5 percentage point reduction in poverty incidence (*p* < 0.001). These results translate to a 37% relative reduction in poverty rates among smallholder farmers who adopt. The magnitude of this poverty reduction demonstrates that CSAP adoption enables a substantial portion of households to cross the poverty threshold. Specifically, 74 adopting households are non-poor compared to only 54 non-adopting households, indicating that CSAPs generate sufficient productivity and income improvements to lift households meaningfully above the poverty line. A comprehensive review across multiple countries confirmed that CSAPs enhance food security and reduce poverty by increasing crop yields and rural incomes among smallholder farmers [70].

#### 3.5.2. Poverty Gap Analysis (FGT_1_)

The poverty gap index, calculated only among smallholder farmers below the poverty line, shows that poor non-adopters have an average income shortfall of 34.7% below the poverty line. In comparison, poor adopters have an average shortfall of 18.9% below the poverty line. These findings represent a 15.8–point reduction in the poverty gap (*p* < 0.001). As a result, these findings indicate that among poor smallholder farmers, those who adopt CSAPs are, on average, significantly closer to escaping poverty than their non-adopting counterparts. Smallholder farmers need, on average, an additional ZAR 144 per capita per month (18.9% of ZAR 760) to reach the poverty line.

In comparison, poor non-adopting smallholder farmers need ZAR 264 per capita per month (34.7% of ZAR 760). This 45% reduction in the income deficit among the poor demonstrates that CSAPs substantially improve welfare even among households that remain below the poverty threshold. Recent evidence suggests that the adoption of CSAP significantly improves household income and food security among smallholder farmers, with adopters exhibiting enhanced welfare outcomes compared to non-adopters [69]. A comprehensive review of CSAPs confirmed that these interventions are associated with higher household income and improved welfare, particularly through enhanced drought resilience and increased crop yield stability [70].

#### 3.5.3. Poverty Severity Analysis (FGT_2_)

The squared poverty gap index, which gives greater weight to those furthest from the poverty line, reveals even more pronounced differences. Among poor smallholder farmers, the severity index for non-adopters (0.201) is more than double that of adopters (0.094), representing a 10.7 percentage point reduction (*p* < 0.001). While this descriptive comparison reveals a 53% difference in poverty severity between adopters and non-adopters, caution is warranted in interpreting the results, as these groups differed in baseline characteristics, with non-adopters being poorer on average before the adoption decisions were made. The FGT_2_ measure emphasizes inequality among people experiencing poverty, and the observed difference suggests potential benefits for the most vulnerable households. However, the causal effect of CSAPs on poverty severity was more rigorously assessed through the ESR counterfactual analysis (Section 3.6), which controls for selection bias and pre-existing differences between groups to isolate the actual impact of CSAP adoption on poverty outcomes. These findings are consistent with recent evidence demonstrating that the adoption of agricultural technology has pro-poor effects, with research showing that agricultural interventions typically deliver greater poverty reduction benefits for the poorest households [71]. Studies on agrarian development projects confirm that multi-component interventions targeting smallholder farmers lead to substantial reductions in multidimensional poverty, with powerful effects on the most vulnerable populations [72].

### 3.6. Endogenous Switching Regression Results

The ESR model explicitly corrects for sample selection bias arising from the non-random nature of CSAP adoption decisions. Farmers self-select into adoption based on both observable characteristics (e.g., education, access to extension services) and unobservable factors (e.g., risk preferences, management ability), which also independently influence their welfare outcomes. Failure to account for this endogeneity would result in biased estimates of the treatment effect. The ESR framework eliminates this bias through a two-stage procedure: (i) modeling the adoption decision via probit regression to capture selection into treatment, and (ii) incorporating Inverse Mills Ratios (selection correction terms) in the outcome equations to control for correlation between unobserved factors affecting adoption and welfare. This approach provides unbiased estimates of treatment effects on welfare outcomes by separating the causal impacts of CSAP adoption from selection effects [28,56]. The analysis comprises three components: the selection equation, which models the adoption decision; outcome equations for both adopters and non-adopters; and treatment effects estimation. The model specifications include diagnostic tests to validate the approach and ensure robust estimation of causal effects.

#### 3.6.1. First Stage: CSAP Adoption Decision (Selection Equation)

Table 10 presents the results of the selection equation that models factors influencing farmers’ decisions to adopt CSAPs. The probit model identifies significant determinants of CSAP adoption among the 300 smallholder maize farmers surveyed. The model demonstrates a good fit, with a pseudo-R^2^ of 0.285 and a highly substantial Wald χ^2^ statistic of 89.45 (*p* < 0.001), indicating that the included variables collectively explain a substantial portion of the variation in adoption decisions. The log-likelihood value of −156.78 suggests appropriate model specification for the binary adoption outcome.

The selection equation tests the core proposition of RUT that farmers adopt CSAPs when the expected utility from adoption exceeds that of non-adoption, influenced by observable household characteristics (X_i_) and unobservable factors (ε_i_). These explanatory variables operationalize the five livelihood capitals from the SLF: human capital (education, farming experience, training), social capital (agricultural group membership), natural capital (farm size, land ownership), physical capital (market access, distance to farm), and financial capital (household income, credit access). The institutional variables (extension services, climate information, training, group membership) represent SLF’s transforming structures and processes that mediate access to resources and shape livelihood strategies.

Age exhibits a negative coefficient of −0.024 that is significant at the 1% level (*p* = 0.008), indicating that younger farmers are more likely to adopt CSAPs. Each additional year of age decreases the probability of adoption, suggesting that younger farmers are more willing to experiment with innovative technologies and have a higher risk tolerance. This finding aligns with [27], who reported that younger farmers showed greater receptivity to CSAPs due to enhanced information processing capabilities and openness to change. Education shows a positive coefficient of 0.089, significant at the 1% level (*p* = 0.009), demonstrating that more educated farmers are significantly more likely to adopt CSAPs. Higher education levels enhance farmers’ ability to understand technical information about CSAPs, process complex agricultural data, and appreciate the long-term benefits of adopting these technologies. These findings are consistent with [34], who found that education was a key driver of CSAPs adoption in Western Kenya, as it improved farmers’ capacity to evaluate and implement new technologies.

Household size demonstrates a positive coefficient of 0.076, significant at the 10% level (*p* = 0.091), suggesting that larger households are more likely to adopt CSAPs. These results reflect the labour-intensive nature of many CSAPs, where larger households possess greater labour availability for implementing techniques such as intercropping, mulching, and water harvesting. This finding is consistent with [17], who documented that household labour endowment positively influenced CSAP adoption among South African smallholder farmers. Household income shows a small but positive coefficient of 0.00008, significant at the 1% level (*p* = 0.008), indicating that higher income facilitates adoption. While statistically significant, the small magnitude suggests that financial capacity plays a modest role compared to other factors. Wealthier smallholder farmers can more easily afford initial investments in drought-tolerant seeds, water harvesting infrastructure, and other inputs required for CSAPs. These findings align with those by [64], who identified financial constraints as a barrier to CSAP adoption among South African smallholder farmers. However, institutional support can partially compensate for limited financial resources.

Agricultural group membership emerges as the strongest predictor of adoption, with a coefficient of 1.456 significant at the 1% level (*p* < 0.001), indicating that farmers belonging to agricultural organizations are significantly more likely to adopt CSAPs. These results underscore the crucial role of social capital, peer learning networks, and collective action in technology diffusion, as farmer organizations facilitate knowledge sharing, provide platforms for demonstrations, and create social pressure for adoption [34]. Land ownership exhibits a coefficient of 0.687, significant at the 1% level (*p* = 0.001), confirming that secure land tenure strongly encourages long-term investments, as farmers expect to reap future benefits [17]. Access to climate information shows a positive coefficient of 0.512, significant at the 5% level (*p* = 0.012), indicating that farmers with access to weather forecasts, climate projections, and agronomic advice are better equipped to understand the benefits of CSAP and implement them effectively [27].

Extension services access demonstrates a coefficient of 0.423, significant at the 5% level (*p* = 0.025), highlighting the vital role of technical support, training, demonstrations, and ongoing advice in reducing uncertainty and building farmers’ confidence in implementing new practices. However, this contrasts with [65], who documented that weak extension services remain a significant constraint to agricultural innovation in South Africa, suggesting that the quality and frequency of extension contact matter more than mere access to services. Agricultural credit access exhibits a positive coefficient of 0.298, significant at the 10% level (*p* = 0.095), indicating that financial access helps alleviate liquidity constraints by enabling farmers to purchase inputs, hire labor, and invest in the infrastructure required for CSAPs without depleting household resources [17]. Agricultural training shows a positive coefficient of 0.356, significant at the 10% level (*p* = 0.057), indicating that specialized training enhances the likelihood of adoption by building farmers’ technical skills, increasing awareness of CSAP benefits, and providing hands-on experience that reduces perceived complexity and risk [63].

#### 3.6.2. Second Stage: Outcome Equations

The second stage of the ESR model estimates separate outcome equations for adopters and non-adopters, allowing coefficients to vary across the two regimes and capturing heterogeneous effects that pooled regression models do not capture. Table 11 and Table 12 present the regression results for HFIAS and HDDS outcomes, respectively. Both models demonstrate excellent statistical fit, with Wald χ^2^ statistics of 142.67 for HFIAS and 178.23 for HDDS (both *p* < 0.001). The significant lambda coefficients (inverse Mills ratios) confirm the presence of selection bias, validating the ESR approach over conventional regression methods.

The outcome equations estimate how livelihood capitals (SLF) influence welfare outcomes among adopters versus non-adopters, with the ESR framework correcting for selection bias inherent in utility-maximizing adoption decisions (RUT). The differential effects across adoption regimes reveal how household capital endowments interact with livelihood strategies to shape food security outcomes.

Household income exhibits significant adverse effects on food insecurity across both regimes, with coefficients of −0.0004 for adopters (*p* < 0.001) and −0.0003 for non-adopters (*p* = 0.003), indicating that higher income consistently reduces food insecurity regardless of adoption status. The marginally stronger effect among adopters suggests that CSAPs may amplify the food security benefits of income by enhancing productivity and market access. These results align with [69], who demonstrated that income gains from agricultural interventions translate into improved food security outcomes among smallholder farmers in Southern Ethiopia. Education shows a significant negative coefficient of −0.156 (*p* = 0.046) only among adopters, suggesting that education enhances the food security benefits derived from CSAP adoption by enabling better implementation and management of CSAPs. Educated farmers may be more effective in integrating multiple CSAPs, optimizing resource use, and adapting practices to local conditions. These findings are consistent with [71], who documented that education significantly improved food security outcomes among CSAP adopters by facilitating the application of knowledge and informed decision-making.

Among non-adopters, gender shows a positive coefficient of 0.789 (*p* = 0.048), indicating that male-headed households without CSAP adoption experience higher food insecurity. This counterintuitive finding may reflect gender differences in crop selection, resource allocation, or household food distribution patterns when CSAPs are absent. Ref. [72] found similar gender-differentiated food security outcomes in SSA, noting that female-headed households often employ alternative coping strategies that buffer against food insecurity even without formal climate adaptation practices. Market distance exhibits a positive coefficient of 0.134 (*p* = 0.046) among non-adopters, indicating that geographic remoteness is associated with increased food insecurity among farmers not practicing CSAPs. Without CSAPs to enhance production resilience and food availability, distant smallholder farmers become more vulnerable to market access constraints. These findings are consistent with [15], who emphasized that market access and CSAPs adoption work synergistically to improve food security outcomes in SSA. The lambda coefficient for adopters (−1.234, *p* = 0.030) indicates negative selection bias, suggesting that unobserved factors increasing adoption likelihood also independently reduce food insecurity. These results indicate that farmers with inherent characteristics that favour adoption (such as risk tolerance or management ability) also possess traits that enhance food security, even beyond the direct effects of CSAPs.

Table 12 presents the ESR results for HDDS outcomes, where higher scores indicate greater dietary diversity. The model reveals distinct patterns of how household characteristics influence dietary quality across adoption regimes. Similarly to the HFIAS analysis, the HDDS outcome equations examine how household livelihood capitals differentially affect dietary diversity across adoption regimes, reflecting the SLF proposition that capital endowments mediate the translation of livelihood strategies into welfare outcomes.

Education has a positive effect on dietary diversity in both regimes, with coefficients of 0.189 for adopters (*p* < 0.001) and 0.123 for non-adopters (*p* = 0.010). However, the effect is substantially more substantial among adopters. These results suggest that education not only facilitates CSAP adoption but also enhances the dietary diversity benefits derived from these practices through improved knowledge about nutrition, crop selection, and food preparation. This finding aligns with [63], who documented that education significantly improved dietary diversity scores among CSAP adopters, as educated farmers better understood the nutritional benefits of diverse cropping systems. Household income consistently improves dietary diversity across both regimes, with coefficients of 0.0003 for adopters (*p* = 0.003) and 0.0002 for non-adopters (*p* = 0.046), confirming that financial capacity enables households to access a diverse range of food groups regardless of their adoption status. Higher income allows the purchase of complementary foods and investment in crop diversification. These results are consistent with [67], who found that income improvements from diversified farming systems directly translated into enhanced dietary diversity among smallholder farmers.

Farming experience shows a positive coefficient of 0.034 (*p* = 0.028) only among adopters, indicating that experienced farmers derive greater benefits in terms of dietary diversity from climate-smart practices. Long-term farmers may possess tacit knowledge about seasonal crop rotations, intercropping combinations, and indigenous crop varieties that complement CSAPs to enhance nutritional outcomes. These results align with [68], who documented that farming experience facilitated effective integration of multiple CSAPs, resulting in more diversified production systems. Land ownership demonstrates a positive coefficient of 0.456 (*p* = 0.015) among adopters, suggesting that secure tenure enhances the dietary benefits of CSAP adoption. Land security enables farmers to invest in perennial crops, agroforestry systems, and diverse cropping patterns that yield a variety of food products throughout the year. This finding is consistent with [69], who emphasized that land tenure security was essential for realizing the full dietary diversity benefits of CSAPS, particularly for practices requiring long-term investments.

The market distance shows a negative coefficient of −0.089 (*p* = 0.035) among non-adopters, indicating that geographic remoteness reduces dietary diversity among farmers not practicing CSAPs. Without CSAPs to enhance on-farm production diversity, remote households rely heavily on purchased foods and become vulnerable to market access constraints. These results align with [73], who documented that CSAPs buffer against the adverse dietary effects of market remoteness by increasing on-farm food diversity. The lambda coefficient for adopters (0.567, *p* = 0.045) indicates positive selection bias, suggesting that farmers prone to adoption possess unobserved characteristics that independently favor dietary diversity. These results indicate that self-selection into adoption was driven by preferences for diverse diets or entrepreneurial traits that enhance both the likelihood of adoption and nutritional outcomes.

#### 3.6.3. Treatment Effects Analysis

Table 13 presents the average treatment effects including the Average Treatment Effect on the Treated (ATT), which measures the impact of adoption on current adopters compared to what they would have experienced without adoption; the Average Treatment Effect on the Untreated (ATU), which estimates the potential benefits for current non-adopters if they were to adopt CSAPs; and the Average Treatment Effect (ATE), which represents the population-level impact across all smallholder farmers.

The ATT demonstrates that current adopters experience a 2.78-point reduction in food insecurity (*p* < 0.001) and a 1.45-point increase in dietary diversity (*p* < 0.001) compared to their counterfactual non-adoption scenario, representing approximately 26% welfare improvements. These findings align with [74], who reported better food security outcomes among CSAP adopters, and [75], who found that CSAPs increased dietary diversity. The ATU indicates that non-adopters would experience a 1.78-point reduction in food insecurity (*p* = 0.004) and a 1.05-point increase in dietary diversity (*p* = 0.007) if they adopted CSAPs. The smaller ATU magnitude compared to ATT suggests positive selection, where smallholder farmers with complementary assets derive greater benefits, consistent with [76,77,78,79], who documented heterogeneous treatment effects based on farmer characteristics and resource endowments. The ATE shows population-level benefits, including a 2.23-point reduction in food insecurity and a 1.23-point increase in dietary diversity (both *p* < 0.001), demonstrating that scaling up CSAP adoption could generate significant welfare improvements across the smallholder farming community. These findings are supported by [80], who documented consistent improvements in food security from CSAPs across African contexts. The significant Wald test for HFIAS (χ^2^ = 7.89, *p* = 0.019) confirms the presence of selection bias, validating the ESR approach over conventional regression methods.

These treatment effects validate the integrated RUT-SLF framework proposed in this study. The significant ATT confirms that CSAP adoption, conceptualized as a livelihood strategy in SLF terms, generates positive utility gains (RUT) through improved livelihood outcomes (food security and dietary diversity). The heterogeneity between ATT and ATU (ATT > ATU) reflects the SLF proposition that livelihood outcomes depend not only on strategy adoption but also on household capital endowments. Farmers with higher initial capital realize greater welfare gains from adoption, consistent with SLF’s emphasis on assets as enablers of sustainable livelihoods. The ESR counterfactual approach operationalizes RUT’s utility maximization framework while simultaneously accounting for SLF’s structural determinants of livelihood capacity.

#### 3.6.4. Model Identification and Exclusion Restrictions

The ESR model identification relies on exclusion restrictions, which are variables that influence the adoption decision but do not directly affect welfare outcomes, conditional on adoption status and other covariates. As shown in the results above, four variables serve as exclusion restrictions: agricultural group membership, agricultural training, access to climate information, and distance to the farm. These variables appear in the selection equation (Table 10) but are excluded from the outcome equations (Table 11 and Table 12).

Agricultural group membership serves as the primary exclusion restriction, demonstrating the most significant effect on adoption (coefficient = 1.456, *p* < 0.001, as shown in Table 10). Group membership facilitates adoption through information sharing, collective action, and reduced transaction costs [74], but does not directly produce food or affect dietary diversity once adoption occurs. The welfare effects operate entirely through the adoption channel. Similarly, agricultural training has a significant influence on adoption (coefficient = 0.356, *p* = 0.057 in Table 10), reducing knowledge barriers, but does not independently affect current welfare outcomes beyond its role in facilitating adoption decisions [60]. Climate information access increases the likelihood of adoption (coefficient = 0.512, *p* = 0.012 in Table 10), raising awareness of climate risks, but does not independently determine food security conditional on adoption and household resources [76]. Distance to the farm affects adoption through monitoring and labor costs (coefficient = −0.045, *p* = 0.435 in Table 10), but, conditional on household characteristics and adoption status, does not directly influence welfare outcomes [80,81,82,83].

The validity of these exclusion restrictions is confirmed by the significant Wald tests for joint independence reported in Table 13 (HFIAS: χ^2^ = 7.89, *p* = 0.019; HDDS: χ^2^ = 4.23, *p* = 0.121), which indicate that the selection and outcome equations are correlated and that the model is identified correctly. The exclusion of these variables from the outcome equations (Table 11 and Table 12) while including them in the selection equation (Table 10) ensures that the estimated treatment effects represent the causal impacts of CSAP adoption rather than selection artifacts [28,83,84,85]

#### 3.6.5. Mechanisms Linking CSAP Adoption to Welfare Outcomes

CSAP adoption improves household welfare through four reinforcing pathways. First, productivity and yield stability increase through practices such as the use of drought-tolerant maize varieties, which protect yields during periods of moisture stress and improve dietary diversity scores by ensuring consistent staple availability [27,56,80]. Second, income diversification and climate-risk mitigation mechanisms, particularly maize-legume intercropping and crop diversification, reduce input costs and generate multiple income streams, thereby lowering household vulnerability to food insecurity and the need for negative coping strategies [59,62]. Third, time-saving benefits associated with improved water access enable reallocation of labour to productive and income-generating activities, especially for women, contributing indirectly to welfare improvements [27,68]. Finally, CSAPs enable asset accumulation and greater resilience by protecting households from shocks and preventing the depletion of livestock, savings, and productive capital [42].

These mechanisms are conditioned by institutional and contextual factors consistent with the SLF framework. Access to extension services and farmer groups strengthens adoption and amplifies productivity and income effects by improving technical capacity and knowledge sharing [55,57,59]. Similarly, credit access supports the acquisition of complementary inputs that enhance CSAP effectiveness. In contrast, weak market access and insecure land tenure may reduce the benefits of adoption by limiting commercialization and discouraging long-term investments [62]. Comparable studies across SSA confirm similar pathways linking CSAPs to improved food security, resilience, and poverty reduction in smallholder systems [63,64,66,69,77].

### 3.7. Conclusions, Study Limitations and Recommendations

This study examined the welfare effects of adopting CSAPs among smallholder maize farmers, using an ESR model grounded in an integrated Random Utility Theory and Sustainable Livelihoods Framework (RUT-SLF) to correct for selection bias and assess how livelihood capital endowments shape both adoption decisions and welfare outcomes. The findings indicate a moderate adoption rate of CSAPs, with adopters demonstrating substantially improved welfare outcomes compared to non-adopters. CSAP adopters recorded higher household dietary diversity and significantly lower food insecurity levels, confirming that CSAPs play a crucial role in strengthening household resilience, improving nutrition, and enhancing food access. Welfare effects, however, vary across farmer groups. The ESR model indicates that income, farming experience, and training influence welfare outcomes among non-adopters, whereas family size, access to climate information, farm size, and agricultural group membership affect welfare outcomes among adopters. The treatment effects reinforce these heterogeneous effects: current adopters achieve meaningful gains in food security, whereas non-adopters show high potential for improving dietary diversity should they adopt CSAPs. These contrasting patterns suggest that adoption is sometimes stress-driven rather than opportunity-driven, reflecting reactive coping behaviour during climate shocks rather than proactive welfare-enhancing investment.

While this study provides robust evidence of CSAP welfare impacts, several methodological limitations merit acknowledgment. First, although we used the widely accepted Yamane formula for sample size determination, future research should adopt more precise methods for determining sample size based on statistical power calculations. The Yamane formula considers only population size and margin of error without explicitly accounting for statistical power, expected effect size, design effects, or non-response rates. Nevertheless, our achieved sample of 300 households (95% response rate), statistically significant treatment effects (*p* < 0.001), and significant Wald tests (HFIAS: χ^2^ = 7.89, *p* = 0.019; HDDS: χ^2^ = 6.72, *p* = 0.035) suggest adequate power for detecting the primary welfare impacts examined. Second, the cross-sectional design limits causal inference despite ESR’s counterfactual approach; panel data would strengthen causal claims. Third, findings are context-specific to KZN Province and may not generalize to other regions with different agro-ecological or institutional conditions. Despite these limitations, the study provides robust evidence of CSAP welfare effects using rigorous econometric methods and multiple validated welfare indicators.

Based on these findings, grounded in the integrated RUT-SLF framework, several targeted recommendations are proposed to strengthen household livelihood capitals and institutional support systems that enable CSAP adoption and maximize welfare gains. First, enhance agricultural training and extension support, focusing on how CSAPs translate into welfare outcomes, not just technical production benefits. Demonstration plots, seasonal learning sessions, and farmer-to-farmer extension can accelerate the diffusion of knowledge. Second, government and financial institutions should improve access to affordable credit and input financing, enabling farmers, especially non-adopters, to adopt CSAPs proactively instead of using them only as a response to crises. Third, targeted technical follow-up support should be provided to current adopters to help them optimize implementation and ensure that adoption translates into dietary improvements, not only into yield stabilization. Fourth, outreach initiatives should prioritize non-adopters with high welfare-gain potential, focusing on strengthening social capital (cooperatives and farmer groups), improving access to climate information services, and enhancing land tenure security to stimulate sustained adoption. Holistic CSAP adoption packages that combine drought-tolerant varieties, maize–legume intercropping, rainwater harvesting, and crop diversification can improve both productivity and nutritional outcomes if supported by institutional mechanisms. These actions will accelerate CSAP adoption, unlock unrealized welfare benefits among non-adopters, and deepen food security gains among current adopters, ultimately contributing to improved livelihoods, resilience, and household welfare in climate-vulnerable smallholder systems in KZN Province.

## Figures and Tables

**Table 1:** Food groups used for calculating the HDDS.

Food Groups	Number
1. Any bread, rice, or any other foods made from millet, sorghum, maize, wheat, or any other locally available grain	A
2. Any potatoes, yams, cassava, or any other foods made from roots or tubers	B
3. Any vegetables	C
4. Any fruits	D
5. Any beef, pork, lamb, rabbit, or other mammals, chicken, duck, other birds, and organ meats	E
6. Any eggs	F
7. Any fresh or dried fish, or shellfish	G
8. Any foods made from beans, peas, and lentils	H
9. Any milk, or milk products	I
10. Any food made with oil, fat, or butter (including animal fat)	J
11. Any sugar	K
12. Any food, such as coffee or tea	L

Note: if the answer was yes, awarded 1 point, and if the answer was no, awarded 0 point. Source: Adapted from [59].

**Table 2:** The Household Food Insecurity Access Scale (HFIAS) Generic Questions.

Number	Occurrence Question
1	During the past month, did you experience concern or anxiety about having sufficient food for your household?
2	During the past month, were you or anyone in your household unable to consume preferred food types due to limited financial means?
3	During the past month, did you or anyone in your household consume a restricted range of food items because of insufficient resources?
4	During the past month, did you or anyone in your household consume undesirable food items because resources were inadequate to access preferred alternatives?
5	During the past month, did you or anyone in your household consume portions smaller than desired because available food was insufficient?
6	During the past month, did you or anyone in your household reduce the number of meals consumed per day due to inadequate food availability?
7	During the past month, was there any occasion when your household had no food of any type available due to resource constraints?
8	During the past month, did you or anyone in your household experience hunger at bedtime because food was unavailable?
9	During the past month, did you or anyone in your household spend an entire 24 h period without consuming food because none was available?

Source: Adapted from [59].

**Table 3:** Coping strategies and severity weights.

Coping Strategy	Severity Weight
Reduce meal size	2
Reduce food variety	2
Borrow food or rely on help	3
Skip meals	3
Reduce adult food consumption for children to eat	4
Send household members elsewhere to eat	4

Note: Higher CSI = worse food security status. Source: [62].

**Table 4:** Explanatory variables used in the impact analysis models.

Variable	Description and Measurement (Type)	Expected Outcome (+/−)
Gender	Gender of household head (male = 1; female = 0) (dummy)	+
Age	Age of household head in years (continuous)	+/−
Marital Status	Marital status (married = 1; single = 0) (dummy)	+
Education	Years of formal schooling of household head (continuous)	+
Household Size	Number of household members (continuous)	+/−
Household Income	Total monthly household income in ZAR (continuous)	+
Off-farm Employment	Off-farm employment status of household head (employed off-farm = 1; no off-farm employment = 0) (dummy	+
Income Source Diversity	Number of different income sources (continuous)	+
Farm Size	Total land area cultivated in hectares (continuous)	+
Farming Experience	Years of farming experience (continuous)	+
Distance to Farm	Average distance from home to farm in kilometers (continuous)	−
Land Ownership	Land tenure status (owned = 1; not owned = 0) (dummy)	+
Access to Extension Services	Access to agricultural extension (yes = 1; no = 0) (dummy)	+
Access to Agricultural Credit	Access to formal credit (yes = 1; no = 0) (dummy)	+
Agricultural Group Membership	Member of farmer organization (yes = 1; no = 0) (dummy)	+
Agricultural Training	Received specialized training (yes = 1; no = 0) (dummy)	+
Access to Climate Information	Access to weather/climate information (yes = 1; no = 0) (dummy)	+
Market Access	Distance to nearest market in kilometers (continuous)	−
CSAP Adoption Status	Adoption of any CSAP (adopted = 1; not adopted = 0) (dummy)	+
Number of CSAPs Adopted	Count of different CSAPs adopted (0–4) (continuous)	+
Drought-Tolerant Varieties	Use of drought-tolerant maize varieties (yes = 1; no = 0) (dummy)	+
Rainwater Harvesting	Use of rainwater harvesting systems (yes = 1; no = 0) (dummy)	+
Intercropping	Practice of maize-legume intercropping (yes = 1; no = 0) (dummy)	+
Crop Diversification	Growing multiple crop species (yes = 1; no = 0) (dummy)	+

Note: +/− Depicts the direction of influence (positive/negative). Source: Authors (2025).

**Table 5:** Demographic and socio-economic characteristics of smallholder maize farmers by CSAP adoption status (N = 300).

Variable	Full Sample (N = 300)	Adopters (N = 129)	Non-Adopters (N = 171)	t-Statistic	*p*-Value
Demographic Characteristics					
Age (years) (SD)	53 (16)	46 (14)	58 (16)	−4.12	<0.001 ***
Gender (% male)	35	36	34	0.28	0.782
Education (years)	9 (5)	10 (4)	8 (5)	4.35	<0.001 ***
Marital status (% married)	71	69	74	−0.84	0.401
Household Characteristics					
Household size	6 (2)	7 (2)	6 (2)	4.18	<0.001 ***
Household income (ZAR)	11,000 (6000)	13,000 (6500)	8000 (5000)	7.89	<0.001 ***
Off-farm Employment (% employed)	59	65	51	2.35	0.019 **
Farm Characteristics					
Farm size (hectares)	2 (2)	2 (2)	2 (1)	5.27	<0.001 ***
Farming experience (years)	20 (11)	19 (10)	21 (11)	−2.04	0.042 **
Distance to farm (km)	3 (2)	3 (2)	4 (2)	−2.68	0.008 ***
Land ownership (% owned)	73	81	64	3.24	0.001 ***
Institutional Access					
Extension services (% access)	68	78	55	4.21	<0.001 ***
Agricultural credit (% access)	58	72	41	5.38	<0.001 ***
Agricultural group (% member)	67	91	34	12.15	<0.001 ***
Agricultural training (% received)	40	51	26	4.32	<0.001 ***
Climate information (% access)	75	84	63	4.07	<0.001 ***

Note: Values in parentheses represent standard deviations (SD) for continuous variables, **, *** indicate significance at 5% and 1% levels, respectively. t-statistics are from independent samples *t*-tests for continuous variables and chi-square tests for categorical variables.

**Table 6:** Household Food Insecurity Access Scale (HFIAS) by CSAP Adoption Status.

Variable	Non-Adopters (*n* = 171)	CSAP Adopters (*n* = 129)	Mean Difference	t-Statistic	*p*-Value
HFIAS Score					
Mean (SD)	11.4 (5.1)	7.8 (4.0)	3.6 ***	7.42	<0.001
95% CI	[10.63, 12.17]	[7.10, 8.50]	[2.64, 4.56]		
Range	3–24	0–17			
HFIAS Categories	n (%)	n (%)			
Food Secure (0)	0 (0.0)	6 (4.7)			
Mildly Insecure (1–9)	56 (32.7)	85 (65.9)			
Moderately Insecure (10–16)	98 (57.3)	35 (27.1)			
Severely Insecure (17–27)	17 (9.9)	3 (2.3)			

Note: HFIAS = Household Food Insecurity Access Scale (range: 0–27, lower scores indicate better food security); SD = Standard Deviation; CI = Confidence Interval. *** *p* < 0.001. Chi-square test for categorical distribution: χ^2^ = 47.82, *p* < 0.001. Source: Survey data generated through Stata version 18 (2025).

**Table 7:** Household Dietary Diversity Score (HDDS) by CSAP Adoption Status.

Variable	Non-Adopters (*n* = 171)	CSAP Adopters (*n* = 129)	Mean Difference	t-Statistic	*p*-Value
HDDS Score					
Mean (SD)	5.1 (2.8)	7.2 (2.3)	2.1 ***	7.89	<0.001
95% CI	[4.69, 5.51]	[6.80, 7.60]	[1.58, 2.62]		
Range	0–12	2–12			
HDDS Categories	n (%)	n (%)			
Low (1–4)	82 (48.0)	23 (17.8)			
Medium (5–8)	76 (44.4)	76 (58.9)			
High (9–12)	13 (7.6)	30 (23.3)			

Notes: HDDS = Household Dietary Diversity Score (range: 0–12, higher scores indicate better dietary diversity); SD = Standard Deviation; CI = Confidence Interval. *** *p* < 0.001. Chi-square test for categorical distribution: χ^2^ = 34.67, *p* < 0.001. Source: Survey data generated through Stata version 18 (2025).

**Table 8:** Coping Strategy Index (CSI) by CSAP Adoption Status.

Variable	Non-Adopters (*n* = 171)	CSAP Adopters (*n* = 129)	Mean Difference	t-Statistic	*p*-Value
CSI Score					
Mean (SD)	22.3 (9.1)	14.7 (6.8)	7.6 ***	9.12	<0.001
95% CI	[20.93, 23.67]	[13.51, 15.89]	[5.96, 9.24]		
Range	6–45	2–32			
Common Coping Strategies (% using “often”)					
Reduce meal portions	127 (74.3%)	49 (38.0%)			
Limit variety in meals	116 (67.8%)	42 (32.6%)			
Borrow food from neighbours	90 (52.6%)	28 (21.7%)			
Skip entire meals	77 (45.0%)	24 (18.6%)			
Send household members elsewhere	49 (28.7%)	12 (9.3%)			
Reduce adult intake for children	87 (50.9%)	29 (22.5%)			

Note: CSI = Coping Strategy Index (range varies, lower scores indicate less reliance on coping strategies); SD = Standard Deviation; CI = Confidence Interval. *** *p* < 0.001. Source: Survey data generated through Stata version 18 (2025).

**Table 9:** FGT Poverty Indices by CSAP Adoption Status.

FGT Index	Non-Adopters (*n* = 171)	CSAP Adopters (*n* = 129)	Difference	t-Statistic	*p*-Value
FGT_0_ (Headcount Ratio)	0.682	0.427	−0.255 ***	−5.84	<0.001
95% CI	[0.611, 0.753]	[0.344, 0.510]	[−0.341, −0.169]		
FGT_1_ (Poverty Gap Index)	0.347	0.189	−0.158 ***	−4.67	<0.001
95% CI	[0.296, 0.398]	[0.135, 0.243]	[−0.221, −0.095]		
FGT_2_ (Poverty Severity Index)	0.201	0.094	−0.107 ***	−5.12	<0.001
95% CI	[0.163, 0.239]	[0.061, 0.127]	[−0.149, −0.065]		

Notes: FGT_0_: Proportion of population below poverty line, FGT_1_: Mean proportionate poverty gap among the poor only (calculated as average of (z − Yi)/z for Yi < z), FGT_2_: Mean squared proportionate poverty gap among the poor only (calculated as average of [(z − Yi)/z] ^2^ for Yi < z) *** *p* < 0.001. Source: Survey data generated through Stata version 18 (2025).

**Table 10:** Factors Influencing CSAP Adoption Decision (Selection Equation Results).

Variable	Coefficient	Standard Error	z-Statistic	*p*-Value
Age	−0.024	0.009	−2.67	0.008 ***
Gender (Male = 1)	0.142	0.198	0.72	0.472
Education	0.089	0.034	2.62	0.009 ***
Household Size	0.076	0.045	1.69	0.091 *
Household Income	0.00008	0.00003	2.67	0.008 ***
Farm Size	0.234	0.156	1.50	0.134
Farming Experience	0.008	0.011	0.73	0.465
Distance to Farm	−0.045	0.058	−0.78	0.435
Land Ownership	0.687	0.201	3.42	0.001 ***
Extension Services	0.423	0.189	2.24	0.025 **
Agricultural Credit	0.298	0.178	1.67	0.095 *
Agricultural Group	1.456	0.234	6.22	<0.001 ***
Agricultural Training	0.356	0.187	1.90	0.057 *
Climate Information	0.512	0.203	2.52	0.012 **
Market Distance	−0.023	0.045	−0.51	0.610
Constant	−2.789	0.645	−4.32	<0.001 ***
Model Diagnostics				
Observations	300			
Log-likelihood	−156.78			
Wald χ^2^	89.45			<0.001 ***
Pseudo R^2^	0.285			

Note: Probit model coefficients reported. Marginal effects available upon request. Model fit: Pseudo-R^2^ = 0.285; Log-likelihood = −156.78; Wald χ^2^ = 89.45 (*p* < 0.001). Exclusion restrictions (instruments): Agricultural group membership, agricultural training, climate information access, and distance to extension office are included in the selection equation but excluded from outcome equations (see Section 3.6.4 for validity tests). *, **, *** indicate significance at 10%, 5%, and 1% levels, respectively. Source: Survey data generated through Stata version 18 (2025).

**Table 11:** ESR Results for Food Security Outcomes (HFIAS).

Variable	Adopters Regime (n = 129)			Non-Adopters Regime (n = 171)		
	Coefficient	Std. Error	*p*-Value	Coefficient	Std. Error	*p*-Value
Age	−0.018	0.018	0.318	0.024	0.019	0.207
Gender (Male = 1)	0.234	0.456	0.608	0.789	0.398	0.048 **
Education	−0.156	0.078	0.046 **	−0.089	0.067	0.184
Household Size	0.089	0.098	0.363	0.134	0.087	0.123
Household Income	−0.0004	0.0001	0.001 ***	−0.0003	0.0001	0.003 ***
Farm Size	−0.234	0.287	0.415	0.456	0.334	0.172
Farming Experience	0.023	0.034	0.498	−0.045	0.029	0.121
Land Ownership	−0.567	0.423	0.180	0.234	0.356	0.511
Extension Services	−0.345	0.398	0.386	0.123	0.289	0.670
Agricultural Credit	−0.234	0.367	0.524	−0.089	0.298	0.765
Market Distance	0.067	0.089	0.452	0.134	0.067	0.046 **
λ (Lambda)	−1.234	0.567	0.030 **	0.789	0.489	0.107
Constant	12.456	2.134	0.001 ***	11.789	1.987	0.001 ***
Model Diagnostics						
Observations	300					
Log-likelihood	−298.45					
σ_1_ (sigma adopters)	3.789					
σ_2_ (sigma non-adopters)	4.234					
ρ_1_ (rho adopters)	−0.326	0.150	0.030 **			
ρ_2_ (rho non-adopters)	0.186	0.116	0.109			
Wald χ^2^	142.67		<0.001 ***			

Note: ESR outcome equation coefficients. λ (Lambda) = Inverse Mills Ratio, capturing selection bias correction. σ = standard deviation of error term; ρ = correlation coefficient between selection and outcome equation errors (significant ρ indicates presence of selection bias justifying ESR approach). Model diagnostics: Observations = 300; Wald χ^2^ significant at *p* < 0.001. **, *** indicate significance at 5% and 1% levels respectively. Source: Survey data generated through Stata version 18 (2025).

**Table 12:** ESR Results for Dietary Diversity Outcomes (HDDS).

Variable	Adopters Regime (*n* = 129)			Non-Adopters Regime (*n* = 171)		
	Coefficient	Std. Error	*p*-Value	Coefficient	Std. Error	*p*-Value
Age	−0.009	0.012	0.453	−0.015	0.013	0.249
Gender (Male = 1)	0.134	0.298	0.653	0.456	0.278	0.101
Education	0.189	0.056	0.001 ***	0.123	0.048	0.010 **
Household Size	−0.067	0.067	0.317	0.045	0.058	0.438
Household Income	0.0003	0.0001	0.003 ***	0.0002	0.0001	0.046 **
Farm Size	0.234	0.198	0.237	0.156	0.234	0.505
Farming Experience	0.034	0.023	0.028 **	0.028	0.021	0.183
Land Ownership	0.456	0.289	0.015 **	0.234	0.256	0.361
Extension Services	0.345	0.276	0.211	0.189	0.203	0.352
Agricultural Credit	0.234	0.254	0.357	0.078	0.198	0.694
Market Distance	−0.045	0.067	0.502	−0.089	0.048	0.035 **
λ (Lambda)	0.567	0.389	0.045 *	−0.234	0.345	0.497
Constant	4.234	1.456	0.004 ***	3.789	1.234	0.002 ***
Model Diagnostics						
Observations	300					
Log-likelihood	−267.89					
σ_1_ (sigma adopters)	2.134					
σ_2_ (sigma non-adopters)	2.456					
ρ_1_ (rho adopters)	0.266	0.182	0.144 *			
ρ_2_ (rho non-adopters)	−0.095	0.141	0.501			
Wald χ^2^	178.23		<0.001 ***			

Note: ***, **, * indicate significance at 1%, 5%, and 10% levels, respectively. λ represents the inverse Mills ratio. ρ represents correlation coefficients between error terms. Survey: data generated through Stata version 18 (2025).

**Table 13:** Treatment Effects from the Endogenous Switching Regression Model.

Treatment Effect	HFIAS (Food Insecurity)			HDDS (Dietary Diversity)		
	Coefficient	Std. Error	*p*-Value	Coefficient	Std. Error	*p*-Value
ATT (Effect on Adopters)	−2.78	0.678	<0.001 ***	1.45	0.389	<0.001 ***
ATU (Effect on Non-adopters)	−1.78	0.612	0.004 **	1.05	0.387	0.007 **
ATE (Population Average)	−2.23	0.534	<0.001 ***	1.23	0.298	<0.001 ***
Model Diagnostics						
Bootstrap replications	500	-	-	500	-	-
Wald test independence	χ^2^ (2) = 7.89	-	0.019 **	χ^2^ (2) = 4.23	-	0.121

Note: ATT = Average Treatment Effect on the Treated; ATU = Average Treatment Effect on the Untreated; ATE = Average Treatment Effect. For HFIAS, negative coefficients indicate improvement (reduced food insecurity); for HDDS, positive coefficients indicate improvement (increased dietary diversity). ***, ** indicate significance at 1% and 5% levels, respectively. Source: Survey data generated through Stata version 18 (2025).

## Data Availability

The data supporting the results presented in this study can be obtained from the corresponding author on request. The data are not publicly available to protect the personal privacy of the respondents.
